# Reciprocal inhibition of PIN1 and APC/C_CDH1_ controls timely G1/S transition and creates therapeutic vulnerability

**DOI:** 10.21203/rs.3.rs-2447544/v1

**Published:** 2023-01-19

**Authors:** Shizhong Ke, Fabin Dang, Lin Wang, Jia-Yun Chen, Mandar T. Naik, Abhishek Thavamani, Yansheng Liu, Wenxue Li, Nami Kim, Nandita M. Naik, Huaxiu Sui, Wei Tang, Chenxi Qiu, Kazuhiro Koikawa, Felipe Batalini, Xiaodong Wang, John G. Clohessy, Yujing Jan Heng, Galit Lahav, Nathanael S. Gray, Xiao Zhen Zho, Wenyi Wei, Gerburg M. Wulf, Kun Ping Lu

**Affiliations:** 1 Division of Hematology/Oncology, Department of Medicine and Cancer Research Institute, Beth Israel Deaconess Medical Center, Harvard Medical School, Boston, MA 02215, USA; 2 Department of Pathology, Beth Israel Deaconess Medical Center and Cancer Research Institute, Harvard Medical School, Boston, MA 02215, USA; 3 Department of Systems Biology, Harvard Medical School, Boston, MA 02215, USA; 4 Laboratory of Systems Pharmacology, Harvard Medical School, Boston, MA 02215, USA; 5 Department of Molecular Biology, Cell Biology & Biochemistry, Brown University, Providence, RI 02912, USA; 6 Yale Cancer Biology Institute, West Haven, CT 06516, USA; 7 Department of Pharmacology, Yale University School of Medicine, New Haven, CT 06510; 8 Key Laboratory of Functional and Clinical Translational Medicine, Fujian Province University, Xiamen Medical College, Xiamen 361023, China; 9 Data Science & Artificial Intelligence, R&D, AstraZeneca, Gaithersburg, USA; 10 Department of Genetics, Harvard Medical School, Boston, MA 02115, USA; 11 Department of Medicine, Division of Medical Oncology, Mayo Clinic, Arizona, USA; 12 Molecular and Integrative Physiological Sciences, Department of Environmental Health, Harvard T.H. Chan School of Public Health, Boston, MA 02215, USA; 13 Preclinical Murine Pharmacogenetics Facility, Beth Israel Deaconess Medical Center, Harvard Medical School, Boston, MA 02215, USA; 14 Department of Chemical and Systems Biology, Chem-H and Stanford Cancer Institute, Stanford University, Stanford, CA 94305, USA; 15 Departments of Biochemistry & Oncology, Schulich School of Medicine and Dentistry, and Robarts Research Institute, Western University, London, ON N6A 3K7, Canada; 16 These authors contributed equally to this work; 17 Lead Contact

## Abstract

Cyclin-dependent kinases (CDKs) mediated phosphorylation inactivates the anaphase-promoting complex (APC/C^CDH1^), an E3 ubiquitin ligase that contains the co-activator CDH1, to promote G1/S transition. PIN1 is a phosphorylation-directed proline isomerase and a master cancer signaling regulator. However, little are known about APC/C^CDH1^ regulation after phosphorylation and about PIN1 ubiquitin ligases. Here we uncover a domain-oriented reciprocal inhibition that controls the timely G1/S transition: The non-phosphorylated APC/C^CDH1^ E3 ligase targets PIN1 for degradation in G1 phase, restraining G1/S transition; APC/C^CDH1^ itself, after phosphorylation by CDKs, is inactivated by PIN1-catalyzed isomerization, promoting G1/S transition. In cancer, PIN1 overexpression and APC/C^CDH1^ inactivation reinforce each other to promote uncontrolled proliferation and tumorigenesis. Importantly, combined PIN1- and CDK4/6-inhibition reactivates APC/C^CDH1^ resulting in PIN1 degradation and an insurmountable G1 arrest that translates into synergistic anti-tumor activity against triple-negative breast cancer *in vivo.* Reciprocal inhibition of PIN1 and APC/C^CDH1^ is a novel mechanism to control timely G1/S transition that can be harnessed for synergistic anti-cancer therapy.

Mammalian cell division is oriented by sequential activation of proline-directed cyclin-dependent protein kinases (CDKs), whose dysregulation contributes to unchecked proliferation and cancer^1–6^. Progression through the G1 phase of the cell cycle requires CDKs, along with their partnering D-type or E-type cyclins^1–6^. Two major regulators required for G1/S transition are the retinoblastoma (RB) protein and the anaphase-promoting complex (APC/C^CDH1^), an E3 ubiquitin ligase that is activated by CDH1 (encoded in humans by *FZR1*), whose activity is regulated by CDK2 and/or CDK4/6 mediated phosphorylation^7–9^. Cyclin D1/CDK4/6 inactivates APC/C^CDH1^ either directly^8^ or indirectly via phosphorylating RB, thereby triggering E2F-dependent upregulation of Cyclins E/CDK2 and EMI1^9, 10^, to promote cell cycle re-entry. A functional collaboration between APC/C^CDH1^ and RB restrains cell cycle entry, as forced pRB-E2F expression alone is insufficient to drive cell cycle entry, and an additional loss of APC/C^CDH1^ E3 ligase activity is required to trigger proliferation^7^. Moreover, the inactivation of APC/C^CDH1^, but not activation of pRB-E2F, represents the commitment point of no return for cell-cycle entry^7^. However, whether APC/C^CDH1^ activity is further regulated after CDK phosphorylation is not known.

Targeting CDK proteins to block cell proliferation has been validated as an effective anti-cancer therapy^11^. CDK4/6 inhibitors have been approved to treat estrogen receptor-positive (ER+) breast cancer (BC)^12^, but only have limited efficacy in triple-negative breast cancer (TNBC). TNBC, especially *RB*-deficient, is the most aggressive and difficult-to-treat subtype of BC with few targeted therapeutic options^13^, underscoring an urgent need for developing novel therapies.

Pro-directed phosphorylation is further regulated by PIN1-catalyzed *cis-trans* prolyl isomerization, which modulates protein functions, including protein stability, interaction, and activity^14, 15^. PIN1 is overexpressed and correlates with poor outcomes in most human cancers^16, 17^. PIN1 activates numerous oncogenic signaling pathways to drive cancer malignancy and drug resistance^16–20^. As a result, *PIN1* knockout, which has no overt phenotype for half of the lifespan in mice^21^, prevents tumorigenesis induced by oncogenes or tumor suppressors^22–24^. Furthermore, *PIN1*-reducing genetic polymorphisms are associated with reduced risk for cancer in humans^25^.

PIN1 was originally identified as a cell cycle regulator and is essential for mitosis in yeast^26^, and many cell-cycle proteins have been identified as PIN1 binding partners^27, 28^. PIN1 modulates RB by stabilizing phosphorylated RB to promote cell cycle progression^29^. PIN1 also regulates cyclin D1 function at transcriptional and posttranslational stabilization^22, 30^. As such, pharmacologic ablation of PIN1, including using the approved drugs, offers a unique and promising approach to eradicate aggressive cancer^17–19, 31–35^. Notably, most PIN1 inhibitors identified so far not only inhibit PIN1’s catalytic activity but also induce PIN1 degradation^31–33, 35^. However, it is still unknown whether and how PIN1 protein stability is physiologically regulated and how PIN1 inhibitors induce PIN1 degradation. Thus, the special prognostic significance of PIN1 protein levels in tumors and the consistent observation of PIN1 degradation upon pharmacologic inhibition prompted us to investigate a PIN1 ubiquitin ligase. Here we show that APC/C^CDH1^, a cell-cycle inhibitor, and PIN1, a cell-cycle promoter, directly interact and negatively regulate each other in a domain-oriented mechanism, which can be harnessed for cancer therapy.

## Results

### Cell cycle regulator APC/C^CDH1^ is a physiological E3 ubiquitin ligase for PIN1

**Extended Data Fig. 1 |:**
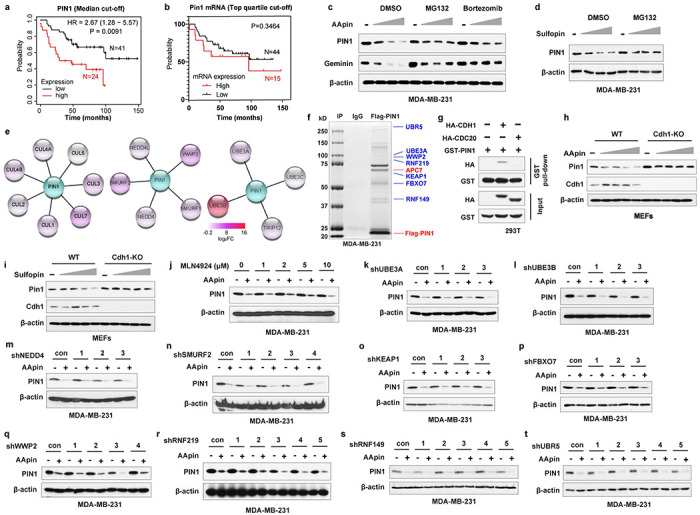
APC/C^CDH1^ is a physiological E3 ubiquitin ligase for PIN1. **a,** Overall survival for BC with low and high PIN1 protein abundance, dichotomized by median expression. Median survival was 13 months for the PIN1-high, but 48 months for the PIN1-low group. **b,** Overall survival for BC with low and high PIN1 mRNA levels, dichotomized by top quartile. **c, d,** IB analysis for indicated proteins derived from MDA-MB-231 cells treated with increasing concentrations of AApin (ATO (0.5, 1, 1.5, 2 μM) plus ATRA (5, 10, 15, 20 μM)) in 1:10 ratio) (c) or Sulfopin (5, 10, 15 μM) (d) for 3 days and 10 μM MG132 or 1 μM Bortezomib for last 12 hrs before harvesting. **e,** Data from PIN1 affinity purification-mass spectrometry experiment was used to generate the interaction networks by Cystoscape. Heatmap represented log_2_-transformed DIA-MS signal changes of GST-PIN1 versus GST. **f,** Coomassie blue stain of Pin1-interacting proteins after immunoprecipitation (IP) resolved by SDS-PAGE. Proteins were identified by mass spectrometric peptide sequencing. MDA-MB-231 cells were transfected with 3XFlag-Pin1 and treated with 10 μM MG132 for 12 hrs. Lysates were immunoprecipitated with M2 (anti-Flag) or control (IgG) beads. **g,** Glutathione S-transferase (GST) pull-down of GST-PIN1 and haemagglutinin (HA)-tagged CDH1. 293T cells were transfected with indicated constructs for 36 hrs and GST-PIN1 were pulled down with glutathione beads. **h, i,** WT or *CDH1* KO MEFs were treated with increasing concentrations of PIN1 inhibitors for 3 days. IB analysis for PIN1. **j,** MDA-MB-231 cells were co-treated with increasing concentration of MLN4924 and AApin (1.5 μM ATO plus 15 μM ATRA) for 3 days. IB analysis of PIN1. **k-t,** Validation of potential PIN1-interacting E3 ligases identified by mass spectrometry. IB of PIN1 from MDA-MB-231 cells stably expressing indicated shRNAs and treated with AApin (1.5 μM ATO plus 15 μM ATRA) for 3 days.

**Fig. 1 |:**
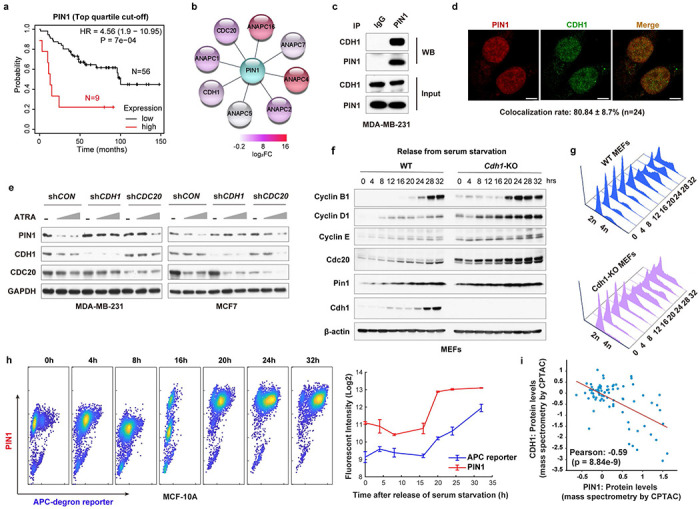
Cell cycle regulator APC/C^CDH1^ is a physiological E3 ubiquitin ligase for PIN1. **a,** Overall survival for BC with low and high PIN1 protein abundance, dichotomized by top quartile expression. Median survival was 13 months for the PIN1-high, but 100 months for the PIN1-low group. **b,** Data from PIN1 affinity purification-mass spectrometry experiment was used to generate the interaction networks by Cystoscape. Heatmap represented log_2_-transformed DIA-MS signal changes of GST-PIN1 versus GST. **c,** Co-IP of endogenous CDH1 with endogenous PIN1. MDA-MB-231 cells were treated with 10 μM MG132 for 12 hrs and precipitated with IgG or anti-PIN1 antibodies. Input is 5% of the total lysates used in IP. **d,** Confocal images of the colocalization of endogenous PIN1 (red) and CDH1 (green). Colocalization rates were calculated by LAS X software. Scale bars, 5 μm. **e,** MDA-MB-231 and MCF-7 cells stably expressing shCDH1 or shCDC20 were treated with increasing concentrations of ATRA (5 μM, 20 μM) for 3 days, followed by IB of cell lysates. **f,** IB of WT and *CDH1* KO MEFs synchronized in G1 phase by serum starvation, followed by releasing back into the cell cycle before harvesting cells at indicated time points. **g,** Cell-cycle profiles corresponding to (f) monitored by fluorescence-activated cell sorting (FACS). **h,** Intensity plots of PIN1 and mCherry-Geminin (aa1-110) reporter in MCF-10A cells synchronized in G1 phase, followed by releasing back into the cell cycle before fixing cells at indicated time points. PIN1 protein was stained with Cy5 (left panel). Fluorescent intensity of PIN1 and Geminin (aa1-110) were quantified and log2 transformed across the time courses (right panel). **i,** Correlation between PIN1 protein levels and CDH1 protein levels across 105 BRCA samples. Protein level z-scores measured with mass spectrometry by the Clinical Proteomic Tumor Analysis Consortium (CPTAC). The graphs were one representative experiment out of three independent experiments. The quantifications of related critical WB were shown in Supplementary Data 2.

PIN1 is a well-established oncoprotein^17^, whose protein, but not mRNA, levels were strikingly correlated with poor prognosis in human BC independent of grade or proliferative indices by analyzing the dataset from the study by Tang et al^36^ (Fig. 1a, Extended Data Fig. 1a, b, **Supplementary Data 1**). Previous studies showed that most PIN1 inhibitors, including the newly developed highly selective Sulfopin^31, 37^, covalent PIN1 inhibitor KPT-6566^35^, and the approved drug combination of ATRA and ATO (AApin)^31, 33^, not only inhibit PIN1’s catalytic activity but also induce PIN1 degradation. As shown^32, 33^, we found that PIN1 inhibitors-induced PIN1 degradation was rescued by proteasome inhibitors (Extended Data Fig. 1c, d), indicating that PIN1 is degraded via the ubiquitin-proteasome pathway. These data suggest that post-translational regulation of PIN1 at the protein level may offer therapeutic opportunities against cancer. We thus investigated the molecular mechanisms controlling PIN1 protein stability.

To identify the specific E3 ubiquitin ligase for PIN1, we used immunoprecipitation coupled with mass spectrometry (IP–MS) and identified potential PIN1-interacting E3 ligases. Based on functional gene similarity, PIN1-interacting E3 ligases identified were categorized into four main groups, with the APC/C E3 ligase complex being the most enriched and validated one in different purification methods (both GST-PIN1 and Flag-PIN1 pull-down) (Fig. 1b, Extended Data Fig. 1e, f**, Supplementary Table 1**). As APC/C activators, CDH1 (encoded by the *FZR1* gene) and CDC20 regulate the activity and substrate specificity of the E3 ligase complex^38^. We found that PIN1 had a much higher affinity for interacting and co-localizing with CDH1 than its close homologue CDC20, as evidenced by immunoprecipitation and immunofluorescence (Fig. 1 c, d, Extended Data Fig. 1g). Moreover, PIN1 inhibitor-induced PIN1 degradation was rescued by knockdown of endogenous *CDH1*, but not of *CDC20* or other candidate E3 ubiquitin ligases identified by our IP-MS in MDA-MB-231 and MCF-7 cells, as well as by knockout (KO) of *Cdh1* in mouse embryonic fibroblasts (MEFs) (Fig. 1e, Extended Data Fig. 1h–t). These results show that CDH1 specifically interacts with PIN1 and likely affects its protein stability.

**Extended Data Fig. 2 |:**
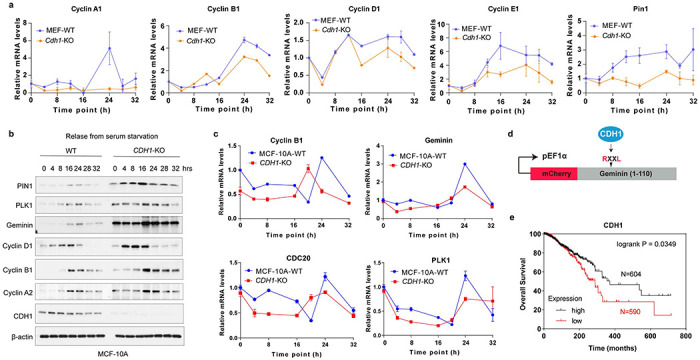
*CDH1* KO stabilizes APC/C^CDH1^ substrates and PIN1 across cell cycle. **a,** RT-PCR analysis of indicated mRNA from WT and *Cdh1* KO MEFs synchronized in G1 phase by serum starvation, followed by releasing back into the cell cycle before harvesting cells at indicated time points. **b,** IB analysis of WT and *CDH1* KO MCF-10A cells synchronized in G1 phase followed by releasing back into the cell cycle before harvesting cells at indicated time points. **c,** RT-PCR analysis of indicated mRNA of WT and *CDH1* KO MCF-10A cells treated as in **b. d,** Schematic diagram of the APC-degron reporter (Geminin: aa1–110). **e,** Overall survival for BRCA tumors in TCGA with low and high *CDH1* mRNA levels.

From the late M phase throughout G1, CDKs inactivation maintains CDH1 in a dephosphorylated state; hence, APC/C^CDH1^ is active, preventing premature entry into S phase^39^. Examining the dynamics of PIN1 protein levels during the cell cycle of two non-transformed cells, we found that PIN1 expression was relatively low in G1 and started to accumulate at the onset of the S phase, coincident with the inactivation of APC/C^CDH1^. Moreover, compared to *CDH1* wild-type (WT), *CDH1* KO in these cells stabilized the protein levels, but not mRNA levels, of mitotic cyclins as well as PIN1 across the cell cycle, revealing a negative correlation between PIN1 protein levels and APC/C^CDH1^ activity (Fig. 1f, g, Extended Data Fig. 2a–c).

To further assess the relationship between PIN1 and APC/C^CDH1^ activity in individual cells at physiological conditions, we used non-transformed MCF-10A stably expressing the Fucci reporter system with mCherry-conjugated to a Geminin fragment (aa1-110) containing the APC/C^CDH1^ degron motif (RXXL)^7^. Notably, the promoter region of the reporter construct is unregulated, and the reporter degradation is primarily regulated by APC/C^CDH1^ (Extended Data Fig. 2d). In this experimental model, an increase in the reporter signal directly reflects a decrease in APC/C^CDH1^ E3 ligase activity and vice versa, allowing for real-time tracking of APC/C^CDH1^ activity at the single cell level^7^. When the cells were synchronized in G1 followed by releasing back into the cell cycle, PIN1 levels were strongly correlated with APC-degron reporter levels across the cell cycle, confirming a negative correlation between PIN1 and APC/C^CDH1^ (Fig. 1h). Moreover, in the CPTAC human breast cancer dataset^40, 41^, we also found that CDH1 protein levels were negatively correlated with PIN1 protein levels. Low levels of CDH1 were associated with poor prognosis of BC tumors (Fig. 1i, Extended Data Fig. 2e). Thus, CDH1 is likely the prime candidate responsible for PIN1 degradation.

**Fig. 2 |:**
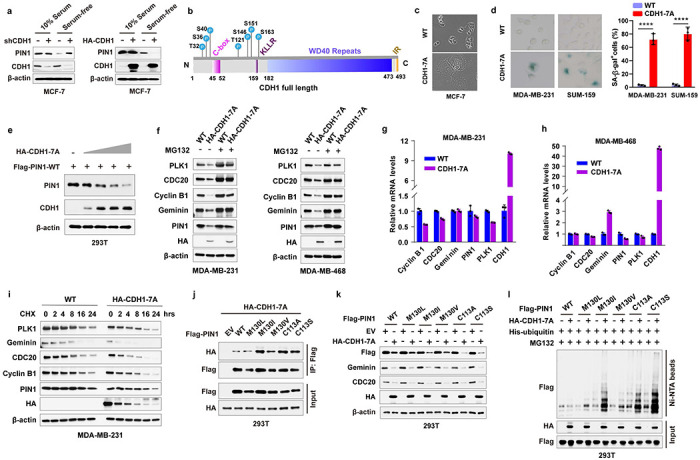
Active APC/C^CDH1^ targets PIN1 for degradation. **a,** IB analysis for indicated proteins derived from MCF-7 cells stably expressing shCDH1 or HA-CDH1 and cultured in 10% serum or serum-free conditions. **b,** Domain architecture of CDH1 contains previously identified serine/threonine sites in the N-terminus that are potential phosphorylation sites for CDKs. The C-box and KLLR motifs are critical for CDH1 association with the APC core complex. The C-terminal IR motif of CDH1 mediates interaction with the TPR subunits APC7 and APC3. **c,** Morphology of MCF-7 stably expressing the CDH1-7A lentiviral construct. **d,** SA-β-gal stain in MDA-MB-231 and SUM-159 cells infected with the CDH1-7A lentiviral constructs. Quantification of SA-β-gal positive cells (percent of total). Data in graphs are mean ± s.d. analyzed by unpaired two-sided t-test. **** P < 0.0001. **e,** IB analysis of indicated proteins derived from 293T cells transfected with indicated constructs. **f,** IB analysis of indicated proteins derived from MDA-MB-231 or MDA-MB-468 cells stably expressing the CDH1-7A lentiviral construct and treated with DMSO or 10 μM MG132 for 12 hrs. **g, h,** RT-PCR analysis of indicated mRNA of WT and CDH1-7A MDA-MB-231 and MDA-MB-468 cells. **i,** CHX chase assay for indicated proteins derived from WT and CDH1-7A MDA-MB-231 cells treated with 50 *μ*/ml CHX for the indicated time. **j,** IB analysis of immunoprecipitates from 293T cells stably co-expressing Flag-PIN1-WT or disabling mutations and the phosphosite-deficient mutant CDH1-7A treated with 10 μM MG132 for 12 hrs and pulled down using Flag-M2 beads. **k,** IB analysis for indicated proteins derived from 293T cells stably co-expressing CDH1-7A or empty vector (EV) in the presence of Flag-PIN1 and its mutants. **I,** IB analysis of the ubiquitinated proteins derived from 293T cells transfected with the indicated constructs for 48 hours and treated with 2 μM MG132 for 12 hrs and pulled down under denaturing conditions by nickel-nitrilotriacetic acid (Ni-NTA) agarose. The graphs were one representative experiment out of three independent experiments.

APC/C^CDH1^ E3 ligase activity is inhibited mainly in cancer cells, likely due to hyperphosphorylation of CDH1 induced by increased CDK kinase activity, resulting in decreased binding to the APC complex^8, 42, 43^. We found that manipulating CDH1 expression affected PIN1 abundance only under serum-free conditions, when CDK activity and CDH1 phosphorylation are relatively low (Fig. 2a). We mutated potential CDK phosphorylation sites that are also potential sites for prolyl isomerization, all of which are located at the N-terminus of CDH1 flanking the C-box (Fig. 2b). Ectopic expression of the phospho-deficient CDH1-7A mutants that can bind the APC core and are constitutively active^43^, induced a senescence-like state, indicative of an irreversible G0 state in several BC cell lines (Fig. 2c, d), and reduced protein levels of PIN1 and other known APC/C^CDH1^ substrates including PLK1, CDC20, Cyclin B1 and Geminin, which was rescued by the proteasome inhibitor (Fig. 2e–f). Notably, the promoter of the Flag-PIN1 construct is unregulated. Thus Flag-PIN1 degradation is primarily regulated by APC/C^CDH1^. As expected, CDH1-7A mutants dramatically shortened the half-lives of its substrates, including PIN1, with limited impact on their mRNA levels (Fig. 2g–i). Furthermore, mutations of the PIN1 active site enhanced the interaction between CDH1-7A and PIN1 and also promoted CDH1-7A-mediated PIN1 degradation and ubiquitination (Fig. 2j–l). Thus, APC/C^CDH1^ is likely the physiological E3 ubiquitin ligase for PIN1, and its activity is primarily inhibited by phosphorylation of CDH1 in cancer cells.

### PIN1 regulates APC/C^CDH1^ E3 ligase activity at post-translational levels to ensure the timely G1/S transition

**Extended Data Fig. 3 |:**
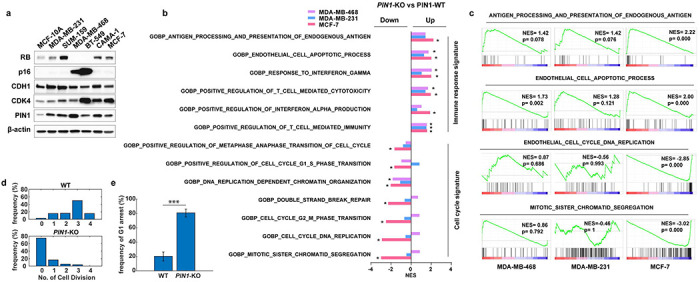
*PIN1* KO restores APC/C^CDH1^ E3 ligase activity **a,** IB analysis for indicated proteins derived from multiple BC cell lines. **b,** Normalized counts of *PIN1* KO versus WT cells were used for GSEA analysis against the biological process related gene sets. Normalized enrichment scores (NES) were used to generate bar graphs for visualization of the functional transcriptional outputs of the three cell lines. *P < 0.001. **c,** Enrichment plots of indicated up-regulated and down-regulated gene sets analyzed by GSEA in *PIN1* KO versus WT cells. **d,** Cell division frequency in WT and *PIN1* KO MCF-7 cells stably expressing the APC-degron reporter from Fig. 1k. **e,** Frequency of G1 arrest (ratio of G1 arrested cells to total cells). The error bar indicates 95% confidence interval determined by bootstrapping. Data in graphs are mean ± s.d., analyzed by unpaired two-sided t-test. ***P < 0.001.

**Fig. 3 |:**
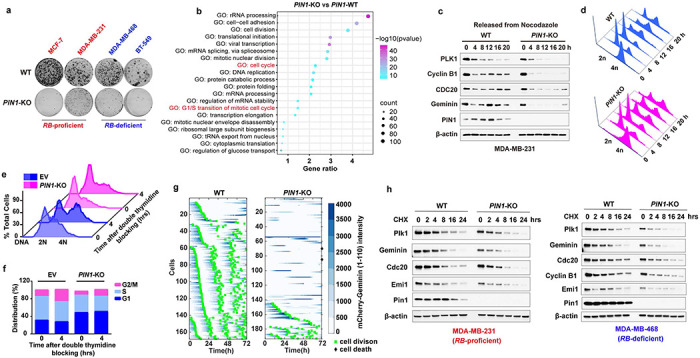
PIN1 regulates APC/C^CDH1^ E3 ligase activity at post-translational levels to ensure the timely G1/S transition. **a,** Long-term colony-formation assay of four BC wild-type and *P1N1* KO cell lines. Cells were grown for about 2 weeks, fixed and stained with crystal violet. **b,** Gene Ontology (GO) enrichment analysis was applied to proteomics of *P1N1* KO versus WT MDA-MB-231 cells. Color codes for p-value (brighter purple is more significant) and symbol size codes for the ratio of proteins related to specific GO term/total number of proteins significantly altered. **c,** IB analysis for indicated proteins derived from WT and *P1N1* KO MDA-MB-231 cells synchronized in M phase by nocodazole and then released back into the cell cycle for the indicated periods of time. **d,** Cell-cycle profiles of WT (blue) and *P1N1* KO (pink) in **c** as determined by FACS. **e,** Release into mitosis after double-thymidine block in the absence or presence of PIN1. DNA contents were measured by FACS in WT and *P1N1* KO MDA-MB-231 cells synchronized at the G1/S boundary by double thymidine block and then released back into the cell cycle for 4 hours. **f,** Cell cycle phase distribution of WT and *P1N1* KO MDA-MB-231 cells from **e. g,** Tracking cell division and cell death on the single cell level. Asynchronous cultures of MCF-7 WT and *P1N1* KO cells expressing the APC-degron reporter were followed for 72 hours for single cell expression of mCherry-Geminin (shades of blue). **h,** Cycloheximide (CHX) chase assay for indicated proteins derived from WT and *P1N1* KO MDA-MB-231 cells (left) or WT and *P1N1* KO MDA-MB-468 cells (right) treated with 50 *μ*g/ml CHX for the indicated time. The graphs were one representative experiment out of three independent experiments. The quantifications of related critical WB were shown in Supplementary Data 2.

Given the negative relationship between PIN1 protein levels and APC/C^CDH1^, to further explore their causal relationship, we knocked out *PIN1* to examine whether PIN1 may reciprocally inhibit APC/C^CDH1^ activity. *PIN1* KO dramatically reduced cell viability in long-term clonogenic assays in both *RB*-proficient and *RB*-deficient BC cell lines (Fig. 3a, Extended Data Fig. 3a). RNA sequencing of three *PIN1* KO BC cell lines revealed a significant positive enrichment of immune response signature across all three *PIN1* KO cell lines, which is consistent with our previous study^31^ and a decreased cell cycle signature in the MCF-7 *PIN1* KO cell line, but not the other two cell lines (Extended Data Fig. 3b, c). In contrast, the proteomic analysis of *PIN1* KO in the MDA-MB-231 cell line showed that *PIN1* KO had noticeable effects on cell cycle progression^33^ (Fig. 3b). Thus, PIN1 may affect cell cycle progression at transcriptional and post-translational levels.

Notably, *PIN1* KO resulted in a prolonged G1 phase, as shown^21^, which was accompanied by de-stabilization of APC/C^CDH1^ substrates across the cell cycle (Fig. 3c–f). To define the effects of *PIN1* KO on APC/C^CDH1^ activation kinetics more precisely in single cells, we analyzed changes in the reporter levels during the cell cycle by tracking MCF-7 wild-type or *PIN1* KO single cells over 72 hours, reporter intensity in asynchronous cultures increased as a result of decreasing APC/C^CDH1^ activity (Fig. 3g, **left panel**, intensity in blue) throughout S phase, leading right up to cell division (green). Notably, *PIN1* KO led to the reactivation of APC/C^CDH1^ along with G1 arrest reflected by starkly decreasing reporter intensity and cell division, or alternatively, prolonged S/G2 phases in some cells, as expected as PIN1 has other substrates in the cell cycle^17^ (Fig. 3g, **right panel,** Extended Data Fig. 3d, e). Of note, it has been shown that PIN1 interacts directly with phosphorylated RB to promote the release of E2F transcription factors^44^, which leads to APC/C^CDH1^ inactivation and cell cycle entry^7^. To separate transcriptional regulation from post-translational, we generated *PIN1* KO in *RB*-proficient and *RB*-deficient cell lines. We found that *PIN1* KO markedly reduced the half-lives of APC/C^CDH1^ substrates independent of RB and transcriptional factors (Fig. 3h), supporting that PIN1 directly regulates APC/C^CDH1^ E3 ligase activity at post-transcriptional levels as well to ensure the G1/S transition.

### Domain-oriented reciprocal inhibition of PIN1 and APC/C^CDH1^ E3 ligase

**Extended Data Fig. 4 |:**
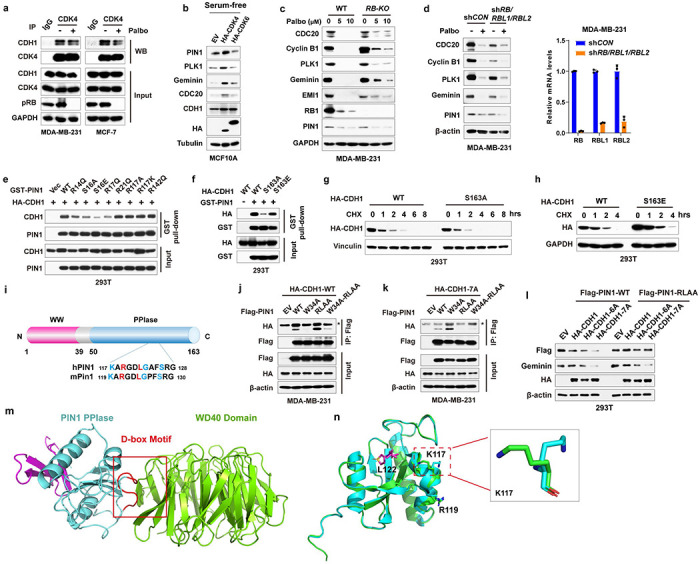
Reciprocal inhibition of PIN1 and APC/C^CDH1^ E3 ligase **a,** Co-IP of endogenous CDH1 with endogenous CDK4. MDA-MB-231 and MCF-7 cells were treated with 1 μM Palbociclib for 24 hrs and precipitated with IgG or anti-CDK4 antibodies. Input is 5% of the total lysates used in IP. **b,** IB analysis of indicated proteins derived from MCF-10A transfected with CDK4 or CDK6 and cultured in serum-free condition for 36 hrs. **c,** IB analysis for indicated proteins derived from WT or *RB* KO MDA-MB-231 cells treated with indicated concentration of Palbociclib for 3 days. **d,** IB analysis for indicated proteins derived from sh*CON* or sh*RB/RBL1/RBL2* MDA-MB-231 cells treated with 5 μM Palbociclib for 3 days. Knockdown of *RB/RBL1/RBL2* was validated by RT-PCR (right). **e,** IB analysis of GST pull-down precipitates derived from 293T cells transfected with HA-CDH1 and GST-PIN1 mutants as indicated for 36 hrs. **f,** IB analysis of GST pull-down precipitates derived from 293T cells transfected with GST-PIN1 and HA-CDH1 mutants for 36 hrs. **g, h,** IB analysis of indicated proteins derived from 293T cells transfected with wild-type CDH1, S163A (g) or S163E (h) mutants CDH1 and treated with 50 *μ*g/ml CHX for the indicated time. **i,** Domain architecture of PIN1 containing an N-terminal WW domain binding specific pSer/Thr-Pro motifs, and a C-terminal peptidyl-prolyl cis/trans isomerase (PPIase) domain that catalyzes prolyl isomerization of specific pSer/Thr-Pro motifs. Note the sequence homology of D-box motifs (RXXL) in human and mouse. **j, k,** IB analysis of immunoprecipitates from MDA-MB-231 cells stably co-expressing Flag-PIN1-WT or disabling mutations in the WW domain (Flag-PIN1-W34A) or the PPIase/D-box domain (Flag-PIN1-RLAA) or the dual mutant (Flag-PIN1-W34A; RLAA), and HA-CDH1-WT (left) or the phosphosite-deficient mutant CDH1-7A (right) treated with 10 μM MG132 for 12 hrs and pulled down using Flag-M2 beads. Non-specific bands for IgG_H_ marked with asterisks (*). **l,** IB analysis of for indicated proteins derived from 293T cells transfected with indicated constructs. The graphs were one representative experiment out of three independent experiments. **m,** A structural modeling illustration of docking the PIN1 PPIase domain (cyan; PDB: 1PIN) to the CDH1-WD40 domain (chartreuse; PDB: 4UI9_R). D-box motif is marked in red. The structure model was generated using HADDOCK. **n,** Superposition of free PIN1 (Green; PDB: 1PIN) and PIN1 in complex with Sulfopin (Cyan; PDB: 6VAJ). The PIN1 PPIase domain in complex with Sulfopin closely resembles free PIN1 with a Root Mean Square Deviation (RMSD) of 0.231 Å. K117 is one of the very few residues that have significant different conformation between free PIN1 and PIN1-Sulfopin complex.

**Fig. 4 |:**
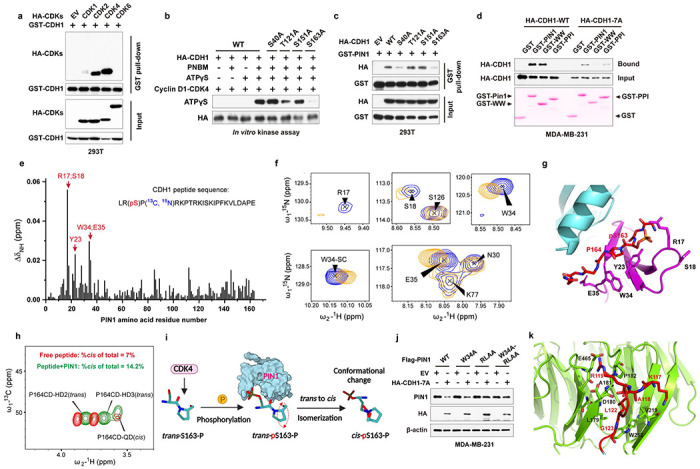
Reciprocal inhibition of PIN1 and APC/C^CDH1^ E3 ligase. **a,** 293T cells were transfected with indicated constructs for 36 hrs. Input is 5% of the total lysates used in IP. **b,** *In vitro* kinase assay showing that CDK4 phosphorylates CDH1 at Ser163. **c,** IB analysis of GST pull-down precipitates derived from 293T cells transfected with HA-CDH1 mutants and GST-PIN1 as indicated for 36 hrs. **d,** GST-PIN1 pull-down precipitates derived from MDA-MB-231 cells stably expressing HA-CDH1 or HA-CDH1-7A, treated with 10 μM MG132 for 12 hrs. The graphs were one representative experiment out of three independent experiments. **e,** NMR analysis of phosphorylated peptide bound to PIN1. Average chemical shift perturbation in PIN1 backbone amide resonances on the binding of the CDH1 phosphopeptide. This data is acquired at pH 6.6 and 25°C with 1:13 molar excess of the CDH1 phosphorylated peptide shown. **f,** Two dimensional (2D) 1H-^15^N Heteronuclear single quantum coherence (HSQC) spectrum of ^15^N-labeled PIN1 protein. The phosphopeptide binding appears fast on the NMR timescale as seen by the movement of representative 15N-HSQC peaks from the backbone of R17, S18, W34, and E35, and the sidechain of W34. **g,** HADDOCK model demonstrating putative interaction between the CDH1 phosphopeptide shown as red sticks and PIN1 WW (magenta) and PPIase domain (cyan; PDB: 1PIN). **h,** Overlay of ^13^C-HSQC spectra acquired on 58 *μ*M free peptide (red) and its 1:4 complex with PIN1 (green). The peak volumes were used to derive isomer population estimates. The graphs were one representative experiment out of two independent experiments. **i,** Schematic diagram illustrating PIN1-catalyzed *trans* to *cis* prolyl-isomerization of the CDH1-pS163-P motif. **j,** IB analysis for indicated proteins derived from MDA-MB-231 cells stably co-expressing phosphosite-deficient CDH1 (CDH1-7A) or empty vector (EV) in the presence of Flag-PIN1 and its mutants. **k,** R119 of PPIase domain forms the electrostatic interaction with CDH1 residues D180 and E465. K117 and G123 of PPIase domain mediate hydrogen bonds with the backbone of V219 and the side chain of W212, respectively. L122 of PPIase domain occupies the hydrophobic pocket formed by CDH1 residues L179, A181 and L467. The quantifications of related critical WB were shown in Supplementary Data 2.

We then investigated the underlying regulation mechanism between PIN1 and APC/C^CDH1^. In the APC/C^CDH1^ complex, CDH1 can be directly phosphorylated and inactivated by Cyclin A2/CDK2, Cyclin D1/CDK4/6, and ERK, resulting in its dissociation from the APC core complex^7, 8^. We found that CDK2 and CDK4, but not CDK6, strongly interacted with CDH1 (Fig. 4a, Extended Data Fig. 4a). Moreover, CDK4 specifically phosphorylated CDH1 at S163 and significantly increased PIN1 and APC/C^CDH1^ substrate abundance in serum-free conditions (Fig. 4b, Extended Data Fig. 4b). A critical target of CDK4/6 is the RB, which binds and inhibits the activating E2F transcription factors. CDK4/6-mediated RB phosphorylation promotes its dissociation from E2Fs and thereby drives the expression of E2F-target genes^45^, including PIN1^46^. To confirm whether CDK4-mediated CDH1 phosphorylation was independent of RB, we knocked down *RB* and its two homologs in BC cells (Extended Data Fig. 4c, d). Although *RB* loss confers a certain level of resistance to CDK4/6 inhibitors, knockdown of *RB* alone couldn’t fully overcome cell cycle arrest induced by CDK4/6 inhibitors^47^. Indeed, consistent with the previous finding, the inhibition of CDK4/6 by relatively higher doses of Palbociclib still potently decreased the levels of PIN1 and other APC/C^CDH1^ known substrates even in *RB*-deficient cells (Extended Data Fig. 4c, d). Thus, these results together support CDH1 as an alternative CDK4 substrate.

PIN1 specifically recognizes pSer/Thr-Pro motifs and catalyzes sequence-specific phosphorylation-dependent proline isomerization^28, 48^. We found that the S->A mutation of CDH1-S163, abolishing phosphorylation at this location, reduced its interaction with PIN1 (Fig. 4c). To gain further insight into the interaction between CDH1 and PIN1 in more mechanistic details, we performed *in vitro* GST pull-down assays of either WT or phosphorylation-deficient CDH1 with full-length GST-PIN1 or its isolated WW or PPIase domain. As expected, CDH1-WT preferentially bound to the PIN1 WW-domain, whereas, surprisingly, the phosphorylation-deficient CDH1-7A preferentially bound to the PIN1 PPIase domain (Fig. 4d), suggesting PIN1 domain-specific interaction modes with CDH1.

To directly visualize PIN1 binding and isomerization of phosphorylated CDH1, we synthesized a CDH1-pS163 peptide and mapped the interaction with PIN1 using nuclear magnetic resonance (NMR). The perturbation data indicated that CDH1-pS163 peptide binds to the WW domain with moderately strong affinity (Fig. 4e). PIN1 residue R17 showed the most substantial perturbation, and along with residues S18, Y23, W34 and E35 formed a continuous patch that interacts with phosphoserine, pS163, and the adjoining proline, P164. Our experiment-guided model suggests that the phosphate group from pS163 has a charge: charge interaction with R17, while P164 stacks in the pocket formed by Y23 and W34 (Fig. 4f, g), which was confirmed by GST pull-down assays using PIN1 point mutations (Extended Data Fig. 4e). Interestingly, weak perturbation was observed in the PPIase domain active site, which may mediate PIN1-catalyzed isomerization of CDH1-pS163 peptide. To confirm such isomerization, we used specific 13C enrichment of the P164 and a 2D-13C HSQC spectrum to directly measure the P164 isomerization states. Our results showed that 7% *cis*-P164 isomer was present in the free peptide, but PIN1-catalyzed isomerization doubled this population to 14.2%, indicative of the *trans* to *cis* isomerization. This might lead to an increase in phosphorylated CDH1 because CDH1-specific phosphatase does not engage with *cis* proline^49^. Indeed, our mutational analysis showed that the S->A phospho-deficient mutation of CDH1-S163 abolished the binding to PIN1 WW domain and became unstable, whereas the S ->E phosphomimic mutation of CDH1-S163 restored the binding to PIN1 and became more stable (Fig. 4h, Extended Data Fig. 4f–h). Thus, PIN1 binds to and catalyzes the *trans* to *cis* prolyl-isomerization of the pS163-P motif in CDH1, thereby stabilizing phosphorylated CDH1 and rendering APC/C^CDH1^ inactive (Fig. 4i).

Distinct from CDH1-WT, phosphorylation-deficient CDH1-7A mutant preferentially bound to the PIN1 PPIase domain, which may mediate PIN1 degradation. To explore this possibility, we examined whether PIN1 contains a destruction box (D-box) since most APC/C^CDH1^ substrates include a D-box with the conserved consensus RXXL sequence (X presents any amino acid)^50^. Indeed, PIN1 has a putative D-box within its PPIase domain (Extended Data Fig. 4i), recognizable by the CDH1 WD40 domain. To confirm that, we performed co-IPs using a point mutation W34A in the PIN1 WW domain and a D-box mutation, RLAA, in the PIN1 PPIase domain and a dual mutation. Indeed, the W34A mutation that is unable to bind to a pSer/Thr-Pro motif^17^ prevented the PIN1 interaction with CDH1-WT. In contrast, the RLAA mutation within the PPIase domain did not interfere with the interaction with CDH1-WT (Extended Data Fig. 4j). However, PIN1-W34A still interacted with CDH1-7A, indicative of a phosphorylation-independent interaction. Only RLAA mutations in the PIN1 PPIase domain prevented the PIN1 interaction with CDH1-7A (Extended Data Fig. 4k), demonstrating a D-box-mediated interaction. In keeping with these findings, the RLAA mutation in PIN1, but not the W34 mutation, conferred resistance to CDH1-7A-mediated PIN1 degradation (Fig. 4j and Extended Data Fig. 4l). Thus, active CDH1 targets PIN1 for degradation via the D-box in the Pin1 PPIase domain.

To gain structural insight into the interaction between the D-box of PIN1 and the WD40 domain of CDH1, we generated a docking model of the complex using HADDOCK (Extended Data Fig. 4m), based on the available structures of PIN1 (PDB: 1PIN)^51^ and the WD40 domain of CDH1 (PDB: 4UI9_R)^52^. Molecular modeling suggests that the formation of electrostatic interaction between R119 of the PPIase domain with CDH1 residues D180 and E465 drives a conformational change of the second β-strand of the PIN1 PPIase domain, leading to L122 of the PPIase domain swinging in the hydrophobic pocket formed by CDH1 residues L179, A181 and L467. Residues K117 and G123 of the PPIase domain-mediated hydrogen bonds with the backbone of V219 and the side chain of W212, respectively (Fig. 4k). These modeling results further support that the D-box in the PIN1 PPIase domain is critical for CDH1 to interact with PIN1 and target PIN1 for degradation. Notably, K117 is one of the very few residues with significantly different conformation between free PIN1 and PIN1-Sulfopin complex (Extended Data Fig. 4n), which may enhance PIN1 interaction with CDH1 to promote PIN1 degradation.

Collectively, the above data show two distinct modes of the PIN1-CDH1 interaction in a domain-oriented manner. On the one hand, when phosphorylated in cells at the G1/S transition, CDH1 binds to the WW domain of PIN1, which catalyzes *trans* to *cis* isomerization of the pS163-P motif in CDH1 to prevent CDH1 dephosphorylation, thereby rendering APC/C^CDH1^ inactive to promote S-phase entry. On the other hand, when unphosphorylated in cells in the G1 phase, CDH1 is active and recognizes the D-box motif in the Pin1 PPIase domain, targeting Pin1 for degradation to prevent S-phase entry. Thus, CDH1 is either a downstream substrate of PIN1 or its upstream regulator, depending on the domain-oriented binding modes to control the timely G1/S transition.

### Pharmacologic inhibition of PIN1 and CDK4 restores APC/C^CDH1^ E3 ligase activity inducing an insurmountable G1 arrest

**Extended Data Fig. 5 |:**
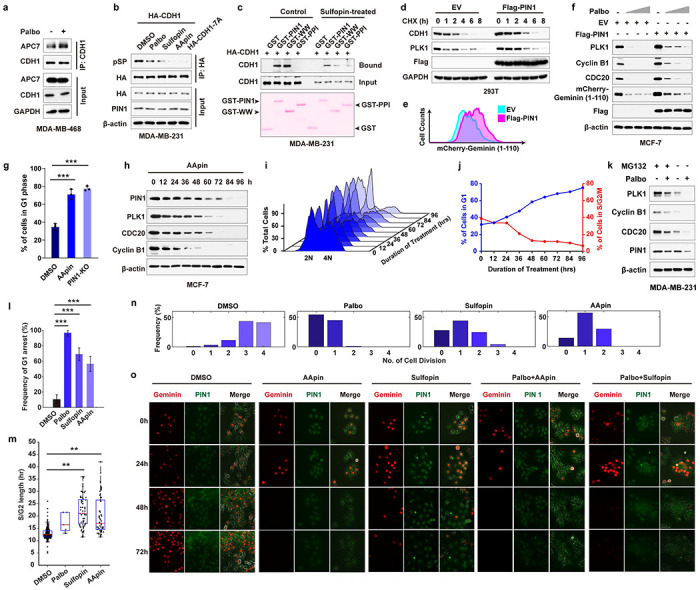
Pharmacologic inhibition of PIN1 and CDK4 restores APC/C^CDH1^ E3 ligase activity inducing an insurmountable G1 arrest. **a,** IB analysis of immunoprecipitates derived from MDA-MB-468 cells treated with 5 μM Palbociclib and pulled down by anti-CDH1 antibody. Input is 5% of the total lysates used in IP. **b,** IB analysis of immunoprecipitates derived from MDA-MB-231 cells stably expressing HA-CDH1 treated with Palbociclib or PIN1 inhibitors and pulled down by anti-HA antibody. HA-CDH1-7A expressed MDA-MB-231 cells were used along with a control. **bc,** IB analysis of indicated GST pull-down precipitates from MDA-MB-231 cells stably expressing HA-CDH1 and treated with vehicle or 10 μM Sulfopin for 3 days and 10 μM MG132 for the last 12 hrs before harvesting. **d,** CHX chase assay for indicated proteins derived from 293T cells transfected with EV or Flag-PIN1 constructs for 36 hrs and treated with 50 *μ*g/ml CHX for the indicated time. **e,** APC-degron reporter levels in MCF-7 cells stably expressing EV or wild-type PIN1 as determined by FACS. **f,** IB analysis for indicated proteins from MCF-7 cells stably co-expressing the APC-degron reporter (mCherry-Geminin (1-110)) and empty vector (EV) or Flag-PIN1 and treated with increasing concentrations of Palbociclib (0.5, 1, 2 μM) for 48 hours. **g,** Percentage of cells in G1 from Fig. 5d. Data in graphs are mean ± s.d., analyzed by unpaired two-sided t-test. ***P < 0.001. **h,** Time course cell cycle regulator expression in MCF-7 cells treated with AApin (10 μM ATRA plus 1 μM ATO). **i, i,** DNA histogram in time course in MCF-7 cells from **h** and quantification of cells in G1 and S/G2/M. The graphs were one representative experiment out of three independent experiments. **k,** IB analysis of indicated proteins from MDA-MB-231 cells treated with vehicle or 1 μM Palbociclib for 3 days and 10 μM MG132 for the last 12 hrs before harvesting. **l,** Distributions of S/G2 duration in DMSO-, Palbociclib- and PIN1 inhibitors-treated MCF-7 cells stably expressing the APC-degron reporter from Fig. 5e. **m,** Frequency of G1 arrest in MCF-7 cells from Fig. 5e (ratio of G1 arrested cells, the last 24 hours, to total cells). The error bar indicates 95% confidence interval determined by bootstrapping. Data in graphs are mean ± s.d., analyzed by unpaired two-sided t-test. **P < 0.01, ***P < 0.001. **n,** Cell division frequency in MCF-7 cells from Fig. 5e. **o,** Immunofluorescence for MDA-MB-231 cells stably expressing the APC-degron reporter (mCherry-Geminin fusion protein) and PIN1 (Venus-PIN1 fusion protein) and treated with different inhibitors for indicated periods.

**Fig. 5 |:**
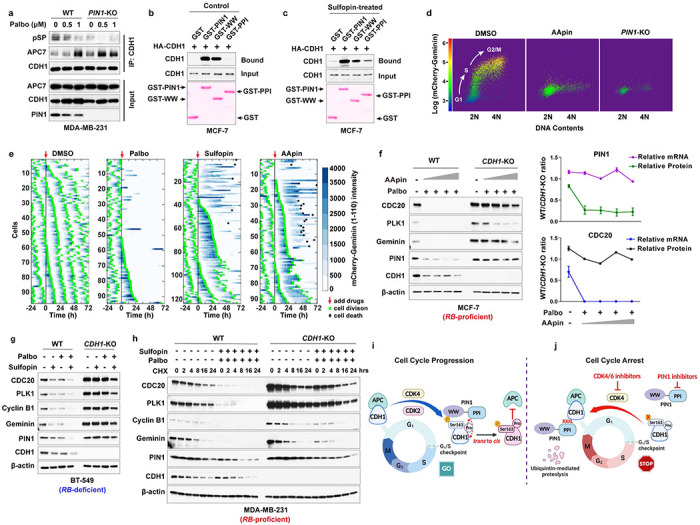
Pharmacologic inhibition of PIN1 and CDK4 restores APC/C^CDH1^ E3 ligase activity inducing an insurmountable G1 arrest. **a,** IB analysis of immunoprecipitates derived from WT and *P1N1* KO MDA-MB-231 cells treated with Palbociclib and pulled down by anti-CDH1 antibody. Input is 5% of the total lysates used in IP. **b, c,** IB analysis of indicated GST pull-down precipitates derived from MCF-7 cells stably expressing HA-CDH1 and treated with vehicle or Sulfopin for 3 days and 10 μM MG132 for last 12 hrs before harvesting. **d,** Dot plots of FACS for WT or *PIN1*-KO MCF-7 cells stably expressing the APC-degron reporter (mCherry-Geminin fusion protein) and treated with DMSO or AApin (1.5 μM ATO plus 15 μM ATRA) for 3 days. DNA were labeled with Hoechst live cell dye. Y-axis, mCherry-Geminin; X-axis, DNA; color code of dots, reporter levels. **e,** Tracking cell division and cell death in response to Palbociclib (4 μM), Sulfopin (10 μM) or AApin (1.5 μM ATO plus 15 μM ATRA) on the single cell level. Asynchronous cultures of MCF-7 cells expressing the APC-degron reporter were followed for 4 days (96 hours) for single cell expression of mCherry-Geminin (shades of blue), mitosis (green), cell death (dark rhomboid). **f,** IB analysis for indicated proteins derived from WT and *CDH1* KO MCF-7 cells treated with a combination of 1 μM Palbociclib and AApin (ATO (0.5, 1, 1.5, 2 μM) plus ATRA (5, 10, 15, 20 μM)) for 3 days. **g,** IB analysis for indicated proteins derived from WT and *CDH1* KO BT-549 cells treated with 5 μM Sulfopin, 2.5 μM Palbociclib or their combination for 3 days. The quantifications of related critical WB were shown in Supplementary Data 2. **h,** CHX chase assay for indicated proteins derived from WT and *CDH1* KO MDA-MB-231 cells pre-treated with combination of 10 μM Sulfopin and 1 μM Palbociclib for 36 hours followed by 50 *μ*/ml CHX treatment for the indicated time. The graphs were one representative experiment out of three independent experiments. **i, j,** Schematic diagrams showing the reciprocal inhibition of PIN1 and APC/C^CDH1^ controls cell cycle progression.

The above results not only identify a novel reciprocal inhibitory mechanism of PIN1 and APC/C^CDH1^ to control the timing of the G1/S transition but also suggest a potential novel anti-cancer therapy targeting PIN1 and CDKs to reactivate APC/C^CDH1^ synergistically. We will use CDK4/6 inhibitors instead of CDK2 inhibitors to reactivate APC/C^CDH1^ in our following experiments, as CDK4/6 inhibitors are highly selective and approved by FDA. To test the possibility, we first examined the effects of *PIN1* KO on the fate of phosphorylated CDH1. *PIN1* KO dramatically reduced CDH1 phosphorylation and promoted the binding of CDH1 to APC complex, which was enhanced by Palbociclib in both *RB*-proficient and *RB*-deficient cells (Fig. 5a, Extended Data Fig. 5a). Similarly, PIN1 inhibition, *i.e.*, prevention of *trans* to *cis*-isomerization, led to dephosphorylation of CDH1 (Extended Data Fig. 5b), presumably by the *trans*-selective CDH1 phosphatase^49^, to initiate the domain-oriented binding mode, in which CDH1 reduced its binding to the PIN1 WW domain, but increased its binding to the PIN1 PPIase domain (Fig. 5b, c, Extended Data Fig. 5c). This change of the binding mode might switch PIN1 from being an upstream regulator to a downstream substrate of CDH1. To support this possibility, we first assessed the impact of PIN1- and CDK4-inhibition on APC/C^CDH1^ activity. Indeed, overexpression of PIN1 potently inhibited APC/C^CDH1^ E3 ligase activity, as determined by the prolonged half-lives and elevated levels of APC^CDH1^ substrates in the absence or presence of the CDK4 inhibitor (Extended Data Fig. 5d–f). By contrast, PIN1 inhibitors led to significant decreases in PIN1 and Geminin along with other APC/C^CDH1^ substrates in a time-dependent manner., corresponding to an increase of G1 cells (Fig. 5d, Extended Data Fig. 5g–j). To maximize the effects of PIN1 inhibitors on PIN1 degradation, *i.e.*, dramatic PIN1 degradation, we applied a 3-day treatment for *in vitro* experiments.

To further confirm these results, we measured APC/C^CDH1^ activation kinetics in single cells upon the CDK4 or PIN1 inhibitors treatment by analyzing changes in the degron reporter levels. CDK4 inhibition caused activation of APC/C^CDH1^ as evidenced by starkly decreased reporter intensity and cell cycle arrest. Only a few cells, presumably those that had already passed the G1/S checkpoint when Palbociclib was administered, proceeded to complete another round of cell division after adding the CDK4 inhibitor, and none had multiple divisions (Fig. 5e, **middle panel**). Hence, CDK4 inhibition likely causes an immediate reactivation of APC/C^CDH1^ reflected by decreased reporter intensity along with G1 arrest rather than cell death. Consistently, proteasome inhibition blocked Palbociclib-induced degradation of APC/C^CDH1^ substrates (Extended Data Fig. 5k). Of note, both PIN1 inhibitors (Sulfopin and AApin) led to prolonged S/G2 phases and cell death in some cells, but eventually a reactivation of APC/C^CDH1^ to induce G1 arrest (Fig. 5e, **right panel,** Extended Data Fig. 5l–n**, Supplementary Movie 1-4**). Thus, as compared with CDK4 inhibition, PIN1 inhibition has a broader activity to arrest cells both in G1 and during mitosis. Significantly, the combination of the CDK4 inhibitor and the PIN1 inhibitor-induced striking activation of APC/C^CDH1^ and G1 arrest (Extended Data Fig. 5o).

**Extended Data Fig. 6 |:**
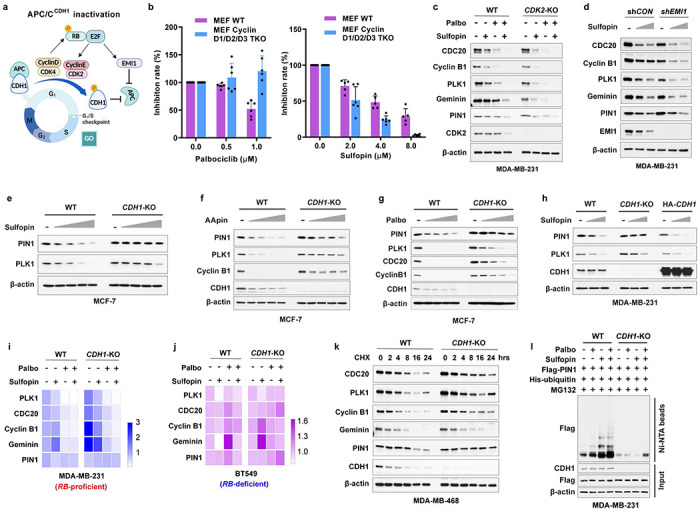
Pharmacologic inhibition of PIN1 and CDK4 restores APC/C^CDH1^ E3 ligase activity inducing an insurmountable G1 arrest. **a,** Schematic diagrams showing the mechanism of APC/C^CDH1^ inactivation by CDKs and EMI1 for cell cycle commitment. **b,** WT and *Cyclin D1/D2/D3* TKO MEFs were treated with Palbociclib or Sulfopin for 4 days and cell viability were assessed by CellTiter-Glo. Data in graphs are mean ± s.d. **c,** IB analysis for indicated proteins derived from sh*CON* and sh*CDK2* MDA-MB-231 cells treated with 10 μM Sulfopin, 0.5 μM Palbociclib or a combination of Palbociclib and Sulfopin for 3 days. **d,** IB analysis for indicated proteins derived from sh*CON* and sh*EMI1* MDA-MB-231 cells treated with increasing concentrations of Sulfopin (5, 10 μM) for 3 days. **e-g,** IB analysis for indicated proteins derived from WT and *CDH1* KO MCF-7 cells treated with increasing concentrations of Sulfopin (2, 4, 8, 10 μM) (e), AApin (ATO (0.5, 1, 1.5, 2 μM) plus ATRA (5, 10, 15, 20 μM)) in 1:10 ratio (f) or Palbociclib (0.5, 1, 2, 4 μM) (g) for 3 days. **i,** RT-PCR analysis of indicated mRNA of WT and *CDH1* KO MDA-MB-231 cells treated with 10 μM Sulfopin, 1 μM Palbociclib or their combination for 3 days. **j,** RT-PCR analysis of indicated mRNA of WT and *CDH1* KO BT-549 cells treated with 5 μM Sulfopin, 2.5 μM Palbociclib or their combination for 3 days. Heatmap represented relative mRNA expression. **k,** CHX chase assay for indicated proteins derived from WT and *CDH1* KO MDA-MB-468 cells treated with 50 *μ*g/ml CHX for the indicated time. The graphs were one representative experiment out of three independent experiments. **l,** IB analysis of ubiquitinated proteins derived from WT and *CDH1* KO MDA-MB-231 cells transfected with the indicated constructs and treated with 1 μM Palbociclib and 10 μM Sulfopin for 3 days and 2 μM MG132 for last 12 hrs and pulled down under denaturing conditions by nickel-nitrilotriacetic acid (Ni-NTA) agarose.

It has been reported that Cyclin D/CDK4 either inactivates APC/C^CDH1^ directly^8^ or indirectly by phosphorylating RB and triggering the onset of E2F-dependent expression of Cyclins E and EMI1, followed by Cyclin E/CDK2- and EMI1-mediated inactivation of APC/C^CDH1^ to commit the G1/S transition^7, 9^ (Extended Data Fig. 6a). Therefore, we examined whether PIN1 inhibitor and CDK4 inhibitor-induced activation of APC/C^CDH1^ is dependent on Cyclin D, RB, CDK2 or EMI1. Knockout or knockdown of each of these genes did not block PIN1 inhibitor- and CDK4 inhibitor-induced activation of APC/C^CDH1^, although triple cyclin D knockout blocked the effects of CDK4 inhibitor (Extended Data Fig. 4c, d, 6b–d). Instead, loss of CDK2 or EMI1 enhanced CDK4 inhibitor- and PIN1 inhibitor-induced activation of APC/C^CDH1^ (Extended Data Fig. 6c, d), further supporting the direct role of PIN1 and CDK4 in the regulation of APC/C^CDH1^. By contrast, knockout of *CDH1* largely rescued PIN1 inhibitor- and CDK4 inhibitor-induced activation of APC/C^CDH1^, as shown by decreases in the protein levels of surrogate markers such as Cyclin B1 and Geminin as well as PIN1 in both *RB*-proficient and *RB*-deficient BC cell lines (Fig. 5f, g, Extended Data Fig. 6e–h). As expected, PIN1 and/or CDK4 inhibitors also reduced transcriptional levels of APC/C^CDH1^ substrates in *RB*-proficient MDA-MB-231 cells but not in *RB*-deficient BT-549 cells (Extended Data Fig. 6i, j). We found that WT-to-CDH1-KO protein ratio, but not WT-to-CDH1-KO mRNA ratio significantly decreased upon PIN1- and CDK4/6-inhibitors treatment (Fig. 5f). Thus, these data strongly supported that PIN1 and CDK4 inhibitors reactivate APC/C^CDH1^ through RB-independent, CDH1-dependent mechanisms, and simultaneous loss of *RB* and *CDH1* was sufficient to overcome the synergistic effects induced by PIN1 and CDK4 inhibitors (Fig. 5g). To further separate transcriptional regulation from post-translational one, we measured protein stability of APC/C^CDH1^ substrates by the cycloheximide chase assay. *CDH1* KO significantly prolonged the protein half-lives of PIN1, and APC/C^CDH1^ substrates with or without PIN1 and CDK4 inhibitors treatment (Fig. 5h, Extended Data Fig. 6k). Combining the PIN1 inhibitor with the CDK4 inhibitor caused even more pronounced PIN1 poly-ubiquitination, which was diminished by *CDH1* KO (Extended Data 6l).

These above results not only uncover that APC/C^CDH1^ E3 ligase activity is inhibited by PIN1-catalyzed *trans-to-cis* isomerization of CDH1 after CDKs-mediated phosphorylation but also suggest that, in addition to conventional cell-cycle regulation, PIN1 inhibition blocks *trans* to *cis* prolyl isomerization of phosphorylated CDH1 and cooperates with CDKs inhibitors to reduce CDH1 phosphorylation, leading to re-activation of APC/C^CDH1^. APC/C^CDH1^ activation, in turn targets PIN1 for degradation via its D-box, thereby creating a negative feedback loop between PIN1 and APC/C^CDH1^ and inducing an insurmountable G1 arrest (Fig. 5i, j).

### PIN1 inhibitors synergize with CDK4 inhibitors against TNBC in human cells and xenograft mouse models

Selective CDK4/6 inhibitors have emerged as effective ER+ BC treatment but only have limited efficacy in TNBC^10, 53^. Besides, most patients achieved only partial remissions and eventually experienced disease progression, indicative of primary and secondary resistance mechanisms, such as genetic alterations in the RB signaling pathway^54–56^. The limitation of CDK4/6 inhibitors underscores the urgent need to develop novel strategies to treat TNBC.

**Extended Data Fig. 7 |:**
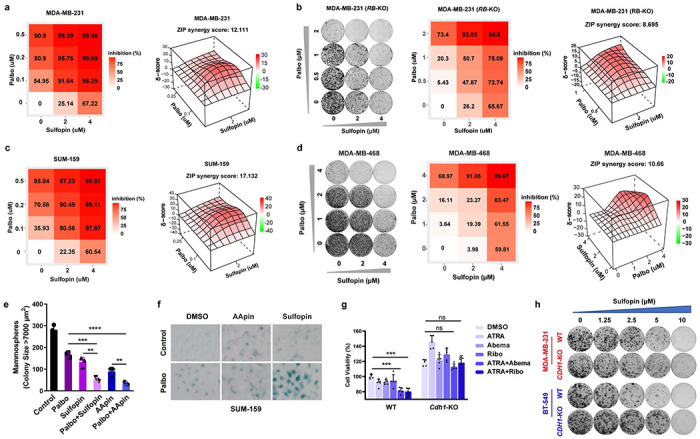
PIN1 inhibitors synergize with CDK4 inhibitors against TNBC *in vitro.* **a,** The heatmap of growth inhibition matrices and synergy scores for Sulfopin in combination with Palbociclib in MDA-MB-231 cells, higher scores (darker red) denoting stronger synergy. **b,** Long-term colony formation assays (left) and growth inhibition matrices (middle) of *RB* KO MDA-MB-231 cells treated with indicated concentrations of Sulfopin and Palbociclib for two weeks. Synergy scores for Sulfopin in combination with Palbociclib in *RB* KO MDA-MB-231 cells (right). **c,** The heatmap of growth inhibition matrices and synergy scores for Sulfopin in combination with Palbociclib in SUM-159 cells. **d,** Long-term colony formation assays (left) and growth inhibition matrices (middle) of MDA-MB-468 cells treated with indicated concentrations of Sulfopin and Palbociclib for two weeks and stained with crystal violet. Synergy scores for Sulfopin in combination with Palbociclib in MDA-MB-468 cells (right). **e,** Mammosphere formation assay of MDA-MB-231 cells treated with 1 μM Palbociclib, 10 MM Sulfopin, AApin (10 μM ATRA plus 1 μM ATO) or a combination of both drugs for two weeks. **f,** Senescence was assessed by staining for SA-β-gal activity for SUM-159 cells treated with AApin (10 μM ATRA plus 1 μM ATO), 10 μM Sulfopin, 1 μM Palbociclib or combinations for 3 days. **g,** WT and *Cdh1* KO MEFs were treated with indicated drugs for 3 days and cell viability were assessed by CellTiter-Glo. Data in graphs are mean ± s.d., analyzed by unpaired two-sided t-test. ***P < 0.001, ns, not significant. **h,** Long-term colony formation assays of WT and *CDH1* KO MDA-MB-231 and BT-549 cells treated with indicated concentrations of Sulfopin for two weeks and stained with crystal violet. The graphs were one representative experiment out of three independent experiments.

**Fig. 6 |:**
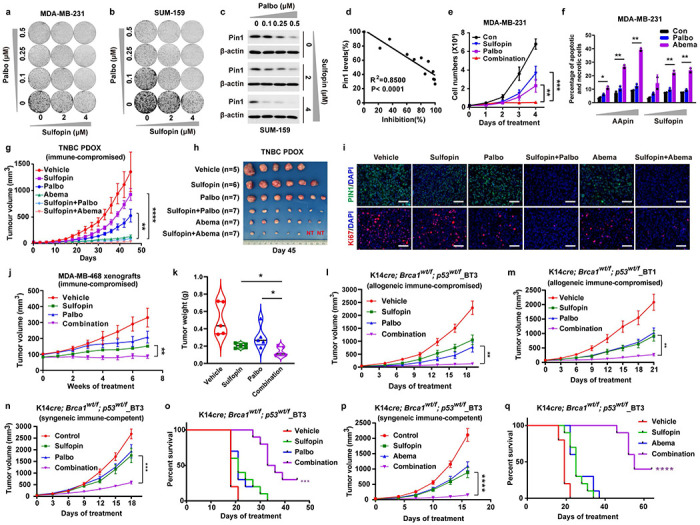
PIN1 inhibitors synergize with CDK4 inhibitors against TNBC *in vitro* and *in vivo*. **a, b,** Colony formation matrices of MDA-MB-231 and SUM-159 cells treated with indicated concentrations of Sulfopin and Palbociclib for two weeks. **c,** IB analysis of PIN1 in SUM-159 cells treated as in **b. d,** Correlation of PIN1 abundance and cell growth inhibition in SUM-159 cells from **b** and **c. e,** Cell counts of MDA-MB-231 cells treated with 1 μM Palbociclib, 10 μM Sulfopin or a combination of both drugs for 4 days. **f,** MDA-MB-231 cells were treated with increasing concentrations of indicated drugs for 3 days, followed by analyzing apoptotic and necrotic cells by Annexin V and PI. Data in graphs are mean ± s.d., analyzed by unpaired two-sided t-test. *P < 0.05, **P < 0.01, ***P < 0.001. **g,** Tumor growth in mice with established TNBC PDOX treated with Sulfopin, CDK4 inhibitors or their combination. **h,** Tumor sizes were measured when mice were euthanized after 45 days. NT, no tumor detectable. Data in graphs are mean ± s.e.m., analyzed by unpaired two-sided t-test. **P < 0.01, **** P < 0.0001. **i,** Representative immunofluorescence images for PDOX tumors stained with PIN1 (green) and Ki67 (red). Scale bars, 50 *μ*m. **j,** Tumor growth in NCG mice with established MDA-MB-468 xenografts treated with Sulfopin, Palbociclib or their combination. **k,** Tumor weights were measured when mice were euthanized after 7 weeks, n=5 mice per group. Data in graphs are mean ± s.e.m., analyzed by unpaired two-sided t-test. *P < 0.05, **P < 0.01. **l,** Growth curve generated from nude mice bearing K14*cre*; *p53wt/f; Brcalwt/f*_BT3 tumors treated with vehicle, Sulfopin, Palbociclib or their combination, n=6 mice per group. **m,** Growth curve generated from nude mice bearing K14*cre; p53wt/f; Brcalwt/f*_BT1 tumors treated with vehicle, Sulfopin, Palbociclib or their combination, n=5 mice per group. **n, o,** Growth curve (n) and survival curve (o) generated from FVB mice bearing K14cre; *p53wt/f; Brcalwt/f*_BT3 tumors treated with vehicle (median survival of 18 days), Sulfopin (median survival of 21 days), Palbociclib (median survival of 21 days) or their combination (median survival of 34.5 days), n=10 mice per group. **p, q,** Growth curve (p) and survival curve (q) generated from FVB mice bearing K14*cre*; *p53wt/f; Brcalwt/f*_BT3 tumors treated with vehicle (median survival of 19 days), Sulfopin (median survival of 25 days), Abemaciclib median survival of 25 days) or their combination (median survival of 55 days), n=10 mice per group. Data are mean ± s.e.m. P values were determined by two-sided unpaired student’s t-test or log-rank test. **P < 0.01, ***P < 0.001, **** P < 0.0001.

Our findings pinpoint CDK4 as one of CDH1 upstream kinases to inhibit APC/C^CDH1^ E3 ligase activity and reveal a reciprocal inhibitory mechanism of PIN1 and APC/C^CDH1^ to control the timely G1/S transition, suggesting potential synergistic effects of combining the PIN1 inhibitors with the CDK4 inhibitors to facilitate the efficacy of CDK4/6 inhibitors, especially in *RB*-deficient tumors. In vitro combination experiments were conducted in multiple TNBC cell lines with matrices of Palbociclib (CDK4 inhibitor) and Sulfopin (highly selective PIN1 inhibitor) to test this possibility. The combination of Palbociclib and Sulfopin resulted in a synergistic antiproliferative effect in both *RB*-proficient and *RB*-deficient TNBC cell lines (Fig. 6a, b, Extended Data Fig. 7a–d). Palbociclib and Sulfopin cooperatively ablated PIN1 protein abundance, which correlated with the antiproliferative effects of this combination (Fig. 6c, d). Similarly, the combination of Palbociclib and Sulfopin or AApin synergistically decreased TNBC cell viability in 3D-organoids (Extended Data Fig. 7e). Consistently, the antiproliferative effects of PIN1- and CDK4-inhibitors were associated with a substantial induction of senescence and apoptosis, as well as a decrease in cell viability in TNBC cell lines (Fig. 6e, f, Extended Data Fig. 7f). However, *CDH1* KO strongly reduced the efficacy of PIN1 and CDK4 inhibition (Extended Data Fig. 7g, h). Altogether, these data suggest that PIN1 inhibitors synergize with CDK4 inhibitors resulting in effective response in TNBC irrespective of RB.

**Extended Data Fig. 8 |:**
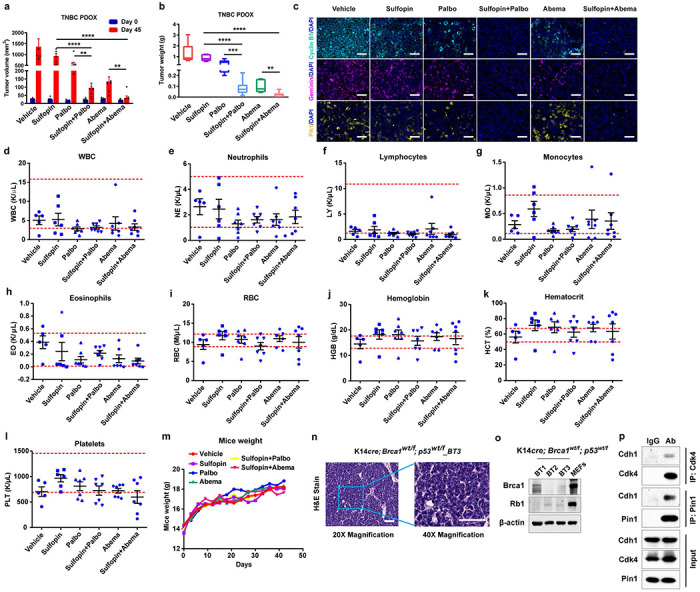
PIN1 inhibitors synergize with CDK4 inhibitors against TNBC in mouse models. **a, b,** Tumor growth in mice with established TNBC PDOX treated with Sulfopin, CDK4 inhibitors or their combination. Tumor sizes (a) and tumor weights (b) were measured when mice were euthanized after 45 days. Data in graphs are mean ± s.e.m., analyzed by unpaired two-sided t-test. **P < 0.01, *** P < 0.001, **** P < 0.0001. **c,** Representative immunofluorescence images for PDOX tumors stained with Cyclin B1 (cyan), Geminin (purple) and PLK1 (yellow). Scale bars, 50 *μ*m. **d-l,** Hematological parameters were analyzed in TNBC PDOX nude mice treated with indicated inhibitors for 45 days. **m,** Body weights were monitored in TNBC PDOX nude mice during the treatments. Data in graphs are mean ± s.e.m. **n,** Representative images of H&E staining in K14*cre; p53wt/f; Brca1wt/f.* Scale bars, 50 μm. **o,** IB analysis for indicated proteins derived from K14*cre; p53wt/f; Brca1wt/f*. tumors or MEFs. **p,** IB analysis of immunoprecipitates derived from K14*cre; p53wt/f; Brca1wt/f* mouse tumors and pulled down by anti-Pin1 or anti-Cdk4 antibody. Input is 5% of the total lysates used in IP.

To further confirm whether our *in vitro* findings can be translated *in vivo* for clinically relevant anti-tumor treatment, we used *RB*-proficient TNBC patient-derived orthotopic xenograft (PDOX) and *RB*-deficient MDA-MB-468 xenograft mouse models to evaluate the anti-tumor efficacy of the two-drug regimen. Although Sulfopin and Palbociclib had limited single-agent efficacy in the *RB*-proficient mouse model, the combination of Sulfopin and the two CDK4 inhibitors nearly entirely suppressed tumor growth, with 2/7 PDOX tumors achieving a complete remission (Fig. 6g, h, Extended Data Fig. 8a, b). These results were supported by immunofluorescent analysis of tumors revealing a significant decrease in the levels of both PIN1 and APC/C^CDH1^ known substrates in tumors from mice treated with Sulfopin and CDK4 inhibitors (Fig. 6i, Extended Data Fig. 8c). It was reported that Abemaciclib inhibits kinases other than CDK4/6 including CDK2^57^, which is also a CDH1 upstream kinase. Therefore, Abemaciclib has the therapy advantage over Palbociclib. As for safety and tolerability, the two-drug regimen showed no bone marrow suppression and was well tolerated with maintenance of body weight (Extended Data Fig. 8d–m). In the *RB*-deficient mouse model, the tumors didn’t significantly benefit from Palbociclib, but the combination of Sulfopin and Palbociclib elicited a complete inhibition of tumor growth (Fig. 6j, k). Thus, PIN1 inhibitors synergize with CDK4 inhibitors against TNBC in human cells and mouse xenograft models.

### PIN1 and CDK4/6 inhibitors achieve synergistic anti-tumor activity against aggressive TNBC in immune-competent mouse models

Next, we thought to confirm their synergistic anti-tumor activity against TNBC in immune-competent mouse models, which is more clinically relevant to human patients. To this end, we generated two cohorts of immune-competent genetically engineered TNBC mouse models: K14*cre; p53wt/f; Brca1wt/f*_BT1 and K14*cre; p53wt/f; Brca1wt/f*_BT3, which were *Rb*-deficient and *Rb*-proficient respectively and resembled aggressive human TNBC with the growth of highly proliferative and poorly differentiated mammary carcinomas in syngeneic immune-competent recipients^58^ (Extended Data Fig. 8n, o**, Supplementary Table 2**).

To ascertain the suitability of our models, we first confirmed the interactions of Cdh1 and Pin1 or Cdk4 in these mouse tumors (Extended Data Fig. 8p). We transplanted the transgenic tumors orthotopically in nude mice or FVB mice to generate allogeneic tumor mouse model (nude mice) and syngeneic tumor mouse model (FVB mice), respectively, Notably, syngeneic BC models are historically much more challenging to treat with chemotherapy or targeted agents than PDOX and allograft models in immune-compromised hosts^59^. Indeed, CDK4 inhibitors or PIN1 inhibitors had limited efficacy in this hard-to-treat, highly undifferentiated, and proliferative murine TNBC. However, their combination was highly effective and well-tolerated and significantly delayed tumor progression and increased overall survival compared to either monotherapy (Fig. 6l–q). Collectively, our results indicate that the combination of PIN1 and CDK4/6 inhibitors achieves synergistic anti-tumor activity against *RB*-proficient or *RB*-deficient TNBC in immune-compromised or immune-competent mouse models, with an excellent safety profile, making it a strong candidate for clinical development.

## Discussion

Epithelial cells execute an active program to maintain interphase that relies on reducing the levels of continuously accumulating pro-mitogenic proteins, including cyclins, through ubiquitin-mediated degradation^1, 60^. A key ubiquitin ligase for the maintenance of interphase is APC/C^CDH1^, which targets a range of pro-mitogenic proteins for degradation^7, 8, 61^. In cancer, APC/C^CDH1^ has been identified as a tumor suppressor^62, 63^. Inactivation of CDH1 is achieved through multi-site Pro-directed phosphorylation^43^, but whether CDH1 is subject to further regulation after phosphorylation is unknown. On the other hand, through isomerization of Pro-directed phosphorylation, PIN1 promotes tumorigenesis by acting as a master cancer signaling regulator activating over 70 oncoproteins and inactivating over 30 tumor suppressors^16, 17^. However, the role and regulation of Pin1 in cell cycle progression remain elusive, even though PIN1 was originally identified as a mitotic regulator^26^.

Here we report for the first time that the two opposing enzymes, APC^CDH1^, a tumor suppressor that promotes maintenance of interphase and can be phosphorylated by CDK4, and PIN1, an oncoprotein that promotes mitotic progression, directly interact with and negatively regulate each other. More importantly, this reciprocal inhibitory mechanism between APC/C^CDH1^ and PIN1 creates a therapeutic vulnerability that can be harnessed to greatly enhance the efficacy of CDK4/6 inhibitors, especially in *RB*-deficient tumors.

In its active, dephosphorylated form, CDH1 binds the D-box of PIN1, which is buried within its PPIase domain and may be exposed by PIN1 inhibitors, and promotes PIN1 ubiquitination-dependent proteolysis. However, when CDK activity garners momentum at the end of G1, CDH1 is phosphorylated specifically at S163, which is the preferred high-affinity binding motif for the PIN1 WW domain. This mediates the PIN1-CDH1 interaction, where PIN1 catalyzes *trans* to *cis* isomerization of the adjacent P164 and reduces CDH1 dephosphorylation by the *trans*-selective CDH1 phosphatase^49^, leading to accumulation of phosphorylated, inactive CDH1 and allowing for S-phase entry. Consequently, the outcome of the PIN1-CDH1 interaction, PIN1 degradation by active CDH1 versus CDH1 inactivation by increased phosphorylation and reduced dephosphorylation due to prolyl-isomerization, depends on CDK4 and PIN1. These activities are tightly regulated in normal cells, but hyperactivated in cancers due to constitutive CDK activation and PIN1 overexpression^17, 64, 65^, resulting in APC/C^CDH1^ inhibition and PIN1 overactivation in a vicious cycle to irreversibly commit cells to overcome the G1/S checkpoint, leading to unchecked cell cycle proliferation^16, 17, 31^.

Three inhibitors of CDK4 are currently approved for the treatment of metastatic BC. As single agent, neither Palbociclib nor Ribociclib has activity in TNBC^66^, while Abemaciclib with a broader target specificity^57^ is under investigation in this setting (NCT03130439). Despite an increased overall survival in Paloma-3 and Monaleesa-2^67, 68^, resistance to CDK4-inhibition inevitably emerges. Despite its mechanism of action, however, RB expression is not associated with and predictive of response in ER+BC^69^ or in TNBC^70^. Hence, more effective CDK4/6 inhibitor combinations for ER+ or TNBC, irrespective of RB status, are urgently needed. Our data show that combined inhibition of CDK4 and PIN1 permits deeper and longer-lasting remissions, even resulting in complete remission in some tumor-bearing mice. Surprisingly, this novel regimen did not appear to affect hematopoiesis, as blood counts remained normal with no detectable toxicity. This is likely due to the fact that both PIN1 and CDKs are tightly regulated during the cell cycle in normal cells. But in cancer, PIN1 is frequently overexpressed and APC^CDH1^ frequently inactivated through phosphorylation, creating a wide therapeutic window for combined inhibition of CDK4/6 and PIN1. Combined inhibition reactivates APC/C^CDH1^ and induces PIN1 degradation, leading to an insurmountable G1 arrest, which translates into synergistic anti-tumor activity against triple-negative breast cancer both in immune-compromised and -competent and/or RB-deficient or -proficient mouse models. Thus, the mechanism we uncovered is important for cancer cell proliferation when PIN1 is overexpressed and CDKs activity is high.

In summary, our work uncovers a novel reciprocal inhibitory mechanism of PIN1 and APC/C^CDH1^ to regulate the G1/S checkpoint, whose aberration causes APC/C^CDH1^ inhibition and PIN1 overactivation in a vicious feedback loop, leading to unchecked cell cycle proliferation and cancer. Moreover, we further develop a new therapeutic strategy using clinically available PIN1 inhibitors and CDK4 inhibitors to break this vicious cycle synergistically to induce anti-cancer activity against not only *RB*-proficient but also *RB*-deficient TNBC, paving the way for new clinical trials to evaluate their clinical impact on patients with TNBC.

## Methods

### Cell lines and plasmids

The human BC cell lines, MDA-MB-231, MDA-MB-468, BT-549, SUM159, MCF-7 and HEK293T were obtained from ATCC. Wild-type and *Cdh1*^−/−^ MEFs were provided by Dr. Wenyi Wei. Among them, MDA-MB-231, MCF-7, HEK293T, MDA-MB-468, BT-549 and MEF cells were cultured in Dulbecco’s modified Eagle’s medium (DMEM) supplemented with 10% fetal bovine serum (FBS). SUM159 cells were cultured in RPMI-1640 medium supplemented with 10% FBS. MCF-10A cells were gifts from the S. D. Cappell group, and cultured in MEBM^™^ Basal Medium and Supplements (Lonza, CC-3150). All the cells used for the experiments were tested negative for mycoplasma contamination.

pLenti-HA-CDH1 was purchased from Applied Biological Materials Inc. pLKO-shRNF219, pLKO-shUBR5, pLKO-shRNF149, pLKO-shSMURF2, pLKO-shWWP2, pLKO-shUBE3A, pLKO-shUBE3B, pLKO-shNEDD4, pLKO-shKEAP1 and pLKO-shFBXO7 were purchased from Sigma-Aldrich. pLenti-HA-CDH1-6A, -7A, pLenti-3X Flag-PIN1, pLenti-3X Flag-PIN1-W34A, -W34A-RLAA, -RLAA, -M130L, -M130I, -C113A and -C113S were generated in our lab. mCherry-Geminin (1-110) and Histone H2B-Turquoise lentiviral vectors were provided by Dr. Jia-Yun Chen. His-ubiquitin constructs was provided by Dr. Yu-Ru Lee. The shRNA library for human E3 ubiquitin ligases (TRC library, RHS4896) was purchased from Thermo Scientific Open Biosystems.

### Reagents and antibodies

ATO (A1010), ATRA (R2625), MG132 (M7449), Thymidine (T1895), Nocodazole (M1404), Glutathione-agarose (G4510), Carboxymethylcellulose sodium salt (CMC-Na, C4888) and Senescence Cells Histochemical Staining Kit (CS0030) were purchased from Sigma-Aldrich. Palbociclib (PD0332991, S1116) and Abemaciclib (S5716) from Selleckchem. Hoechst 33342 Solution and Dead Cell Apoptosis Kit with Annexin V FITC and PI from Thermo. Tumor Dissociation Kit (mouse) from Miltenyi Biotec. Sulfopin was provided by Dr. Nathanael Gray.

Antibodies used in this study are as follows: Anti-PIN1 mouse monoclonal antibody was provided by Dr. Xiao Zhen Zhou. Anti-Cdh1 (sc-56312) and anti-CDC20 (sc-13162) antibodies were purchased from Santa Cruz. Anti-Pin1 rabbit monoclonal antibody (ab192036), Anti-RB (ab181616), anti-APC7 (ab4171), anti-CDK4 antibody (ab68266) and anti-Thiophosphate ester (ab133473) antibodies were purchased from Abcam. Monoclonal anti-Flag M2 antibody (F1804) from Sigma. Anti-HA-Tag rabbit mAb (3724), anti-HA-Tag mouse mAb (2367), anti-CDK2 rabbit mAb (2546), anti-CDK4 (D9G3E) rabbit mAb (12790), anti-Cyclin B1 antibody (4138), anti-PLK1 rabbit mAb (4513), anti-Phospho-MAPK/CDK Substrates (PXS*P or S*PXR/K) (2325) and anti-Geminin rabbit mAb (52508) antibodies were purchased from Cell Signaling Technology. Anti-Emi1 mouse mAb (376600) was purchased from Thermo.

### In gel digestion, mass spectrometry and data procession

MDA-MB-231 cells were lysed in pull-down buffer (20 mM Tris pH 8.0, 150 mM NaCl, 1 mM EDTA pH 8.0, 0.5% Nonidet-P40). The cell extracts were pre-cleared by glutathione agarose beads and incubated with 1 μM GST or GST-PIN1 overnight at 4 °C. Protein complexes were recovered on glutathione agarose beads for 2 hours at 4 °C, washed four to six times with pull-down buffer and eluted by boiling in SDS–containing sample buffer. Bound proteins were resolved by SDS-PAGE on 4-12% gradient gel (Invitrogen) for staining with Coomassie Brilliant Blue R350 (GE Healthcare).

The gel lanes stained with Coomassie blue were unevenly excised into 6 sections. Each section was cut into approximately 1-mm^3^ pieces. The gel slices were first destained with the 30 % acetonitrile in 100 mM NH_4_HCO_3_, dried by speedvac, and then incubated with 10 mM Dithiothreitol (DTT) for 1 h at 56 °C and then 20 mM iodoacetamide (IAA) in dark for 45 min at room temperature. After reduction and alkylation, the samples were digested with trypsin (Promega) at 10 ng/*μ*L overnight at 37 °C. The supernatant was collected and then combined with peptides digested and extracted from the gel slices with 80 % acetonitrile containing 0.1% TFA. Peptide purification was performed on C18 column (MarocoSpin Columns, NEST Group INC) and 1 *μ*g of the peptide was injected for mass spectrometry analysis.

The samples were measured by data-independent acquisition (DIA) mass spectrometry method as described previously^71–73^. The Orbitrap Eclipse Tribrid mass spectrometer (Thermo Scientific) instrument coupled to a nanoelectrospray ion source (NanoFlex, Thermo Scientific) and EASY-nLC 1200 systems (Thermo Scientific, San Jose, CA). A 120-min gradient was used for the data acquisition at the flow rate at 300 nL/min with the temperature controlled at 60 °C using a column oven (PRSO-V1, Sonation GmbH, Biberach, Germany). All the DIA-MS methods consisted of one MS1 scan and 33 MS2 scans of variable isolated windows with 1 m/z overlapping between windows. The MS1 scan range is 350 – 1650 m/z and the MS1 resolution is 120,000 at m/z 200. The MS1 full scan AGC target value was set to be 500 % and the maximum injection time was 100 ms. The MS2 resolution was set to 30,000 at m/z 200 with the MS2 scan range 200 – 1800 m/z and the normalized HCD collision energy was 28%. The MS2 AGC was set to be 4000 % and the maximum injection time was 50 ms. The default peptide charge state was set to 2. Both MS1 and MS2 spectra were recorded in profile mode. DIA-MS data analysis was performed using Spectronaut v16^74–76^ with directDIA algorithm by searching against the Uniprot^77^ downloaded human fasta file. The oxidation at methionine was set as variable modification, whereas carbamidomethylation at cysteine was set as fixed modification. Both peptide and protein FDR cutoffs (Qvalue) were controlled below 1% and the resulting quantitative data matrix were exported from Spectronaut. All the other settings in Spectronaut were kept as Default.

### *In vitro* treatment

Sulfopin and AApin (ATRA+ATO) treatment was described as previously^33, 37^. Briefly, cells were seeded in 6-well plates and treated with increasing concentrations of AApin (ATO (0.5, 1, 1.5, 2 μM) plus ATRA (5, 10, 15, 20 μM) in 1:10 ratio) for 3 days. Cells were treated with increasing concentration of Sulfopin (2, 4, 8, 10 μM) or Palbociclib (0.5, 1, 2, 4 μM) for 3 days. Drugs were replenished in media every 24 hours to ensure that PIN1 and CDK4/6 inhibition was maintained for the duration of the experiment.

### STED imaging

Except where indicated otherwise the steps were performed at room temperature. Cells were rinsed with PBS twice and fixed with 2% PFA for 15 min. Fixative was removed by washing with PBS 3 times. Cells were then permeabilized with 0.1% Triton for 10 min. After removing Triton, cells were blocked with 5% BSA for 1 hour and then incubated with anti-PIN1 (Abcam) and anti-CDH1 (Santa Cruz) antibodies overnight at 4°C. After three washes with PBS, the cells were then incubated with Alexa Fluor^®^ 514 Goat Anti-Mouse (Invitrogen) and Alexa Fluor^®^ 568 Goat Anti-Rabbit (Abcam) antibodies for 1 hour. Following the incubation, the cells were washed 3X with PBS and mounted for STED imaging. Colocalization rates were calculated using the LAS X software (Leica).

### Real-time PCR

Total RNAs were extracted using the QIAGEN RNeasy mini kit. cDNA synthesis was performed using Maxima Universal First Strand cDNA Synthesis Kit from Thermo Scientific. qPCR reactions were performed with FastStart Universal SYBR Green Master (Rox) from Roche. The experiments were performed according to the manufacturer’s instructions. The sequences of the primers used for qRT-PCR analyses were provided in Supplementary Table 3.

### RNA sequencing and data analysis

Total RNAs were extracted from the BC cell lines WT and *PIN1* KO MDA-MB-231, MCF-7 and MDA-MB-468 respectively. RNA-sequencing samples were prepared as previously described^78^. Gene set enrichment analysis (GSEA) was performed using GSEA software (Broad). Normalized counts of *PIN1* KO versus WT cells were used for GSEA analysis against the biological process related gene sets. Normalized enrichment scores (NES) were used to generate bar graphs for visualization of the functional transcriptional outputs of the three cell lines.

### Drug combination test and synergy calculations

MDA-MB-231, MDA-MB-468 and SUM-159 cells were seeded out in appropriate dilutions and treated with increasing concentrations of two drugs to form colonies in 1-3 weeks. Colonies are fixed with methanol (100% v/v), stained with crystal violet (0.5% w/v) and counted using Celigo Image Cytometer. The percentage of growth inhibition was calculated based on colony numbers and areas. The inhibition heatmaps and ZIP synergy scores were generated and calculated by SynergyFinder^79^.

### Time-lapse live imaging and single-cell tracking

MCF-7 cells were stably expressed with mCherry-Geminin (1-110) and a histone H2B-Turquoise. Cells were then plated 24 hours before starting the microscope acquisition. PIN1 inhibitors or CDK4/6 inhibitor were added in the medium and imaged using a Nikon Eclipse TE-2000 inverted microscope with a 10X Plan Apo objective and a Hammamatsu Orca ER camera, equipped with environmental chamber controlling temperature, atmosphere (5% CO2) and humidity. Images were acquired every 30 min using the MetaMorph Software. For each condition filmed, 4 different fields were selected.

p53Cinema single cell analysis package was used for semiautomatic tracking of individual cells in live cell imaging datasets as described previously^80^. Tracking data were then used to quantify intensity of fluorescent reporters from background subtracted images by averaging 10 pixels within the cell nucleus. Cells were tracked using only information about a constitutively expressed nuclear marker, such as H2B-Turquoise, and were thus blind to the dynamics of molecular players of interest, such as mCherry-Geminin. Only cells that remained within the field of view throughout the entire duration of the experiment were considered for downstream analyses. We defined the frequency of G1 arrest as those cells that arrested in G1 phase for at least 20 hours after drugs were added. S/G2 durations were calculated by the time that cells spent in S/G2 phase after drugs were added.

### Cell synchronization and cell cycle profiling

Cells synchronized by double thymidine block or nocodazole block as described previously ^81^ were collected at the indicated time points and suspended in cell cycle kit (Beckman Coulter) according to the manufacturer’s instructions. Stained cells were sorted with CytoFLEX LX1 Flow Cytometer. The results were analyzed by FSC Express software.

### Annexin V-FITC–PI double staining

For detection of apoptosis, cells treated with indicated inhibitors were co-stained with Annexin V-FITC and PI (Dead Cell Apoptosis Kit, Invitrogen) according to the manufacturer’s instructions. Stained cells were sorted with CytoFLEX LX1 Flow Cytometer.

### Immunoblot and immunoprecipitation analyses

For IB analysis, cells were lysed in RIPA buffer (Thermo) supplemented with protease inhibitors (Sigma) and phosphatase inhibitors (Sigma). Protein concentrations were measured using Protein Assay Dye Reagent (Bio-Rad) and a Beckman Coulter. Equal amounts of protein were resolved by SDS-PAGE and probed with indicated antibodies. For immunoprecipitations analysis, cells were lysed in IP lysis buffer (Thermo) and pre-cleared by Mouse IgG-Agarose (Sigma) for 1 hour at 4 °C and then incubated with anti-Flag M2-Agarose (Sigma) for 2 hours at 4 °C. The agaroses were washed four times with IP lysis buffer and boiled in standard Laemmli-Buffer with 5% final concentration of β-mercaptoethanol before being resolved by SDS–PAGE and probed with indicated antibodies.

### GST pull-down assay

GST pull-down was performed as described previously^82^. Briefly, cells were stably expressing indicated proteins and lysed in pull-down buffer (20 mM Tris pH 8.0, 150 mM NaCl, 1 mM EDTA pH 8.0, 0.5% Nonidet-P40). The cell extracts were pre-cleared by glutathione agarose beads and incubated with 1 μM GST or GST fusion proteins overnight at 4 °C. Protein complexes were recovered on glutathione agarose beads for 2 hours at 4 °C, washed four to six times with pull-down buffer and eluted by boiling in SDS–containing sample buffer. Bound proteins were resolved by SDS–PAGE.

### *In vivo* ubiquitination assay

*In vivo* Ubiquitination Assay was performed as described previously^83^. 293T cells were transfected with His-ubiquitin and the indicated constructs. Thirty-six hours after transfection, cells were treated with 2 μM MG132 for 12 hours and lysed in buffer A (6 M guanidine-HCl, 0.1 M Na_2_HPO_4_/NaH_2_PO_4_, and 10 mM imidazole pH 8.0). After sonication, the lysates were incubated with Ni–NTA beads (QIAGEN) for 3 h at 4 °C. Subsequently, the His pull-down products were washed twice with buffer A, twice with buffer A/TI (1 volume buffer A and 3 volumes buffer TI), and once with buffer TI (25 mM Tris-HCl and 20 mM imidazole pH 6.8). The pull-down proteins were resolved by SDS–PAGE for IB.

### *In vitro* kinase assay

*In vitro* kinase assay was performed as previously described^84^. Briefly, HA-tagged CDH1 WT and mutants were transfected into HEK293T cells, followed by being immunoprecipitated with monoclonal Anti-HA-Agarose antibody (Sigma, A2095). The purified HA-CDH1 proteins were then incubated with 500 uM of ATPγS (Abcam, ab138911) and 0.5 ug of recombinant human cyclin D1+CDK4 proteins (Abcam, ab55695) in the kinase reaction buffer (50mM Tris-HCl, 10mM MgCl_2_, 0.1mM EDTA, 2mM DTT, 0.01% Brij 35, pH 7.5) for 30 min at room temperature. Then adding 2 mM of PNBM (Abcam, ab138910) and allowing the alkylating reaction proceed for additional 2h at room temperature. The reaction was then terminated by adding 5x SDS loading buffer and boiled for 10 min. Samples were then subjected to IB using anti-Thiophosphate ester antibody (Abcam, ab92570).

### *In vivo* therapy for patient-derived xenografts

All animal experiments were approved by the IACUC of the Beth Israel Deaconess Medical Center. Triple-negative BC patient-derived xenograft (TM00096) was purchased from Jackson Laboratories. Pieces from PDOX tumors were subcutaneously implanted into the mammary fat pads of 6-week-old BALB/c female nude mice. Tumor sizes were measured every three days by caliper after implantation and tumor volume was calculated by the modified ellipsoidal formula: tumor volume = ½ length × width^2^. Treatments were started once the tumors reached 3-5 mm in diameter and continued until tumors reached 15 mm in any direction. Mice were randomly assigned to six groups with comparable average tumor size. Sulfopin treatment was given by intraperitoneal injection with a dosage of 40 mg/kg (dissolved solution: 5% DMSO in D5W, 7 days/week), Palbociclib treatment was given by oral gavage with a dosage of 100 mg/kg (dissolved solution: saline, 5 days/week), Abemaciclib treatment was given by oral gavage with a dosage of 100 mg/kg (dissolved solution: 0.5% CMC-Na, 5 days/week), or drug combinations in which each compound was administered at the same dose and scheduled as a single agent. The investigators were not blinded to allocation during experiments and outcome assessment.

### *In vivo* therapy for immunocompetent TNBC mouse models

All animal experiments were approved by the IACUC of the Beth Israel Deaconess Medical Center. Maximum permitted the longest dimension of tumors was 20 mm. Pieces from breast tumors generated in K14*cre; p53wt/f; Brca1wt/f* female mice were transplanted into the mammary pads of 6-week-old FVB female mice. For survival studies, treatments were started once the tumors reached 3-5 mm in diameter and continued until mice were symptomatic or tumors reached 20 mm in any direction, at which point mice were euthanized. For time point analysis, mice were sacrificed two weeks post-treatment initiation. Sulfopin treatment was given by intraperitoneal injection with a dosage of 60 mg/kg (7 days/week), Abemaciclib treatment was given by oral gavage with a dosage of 100 mg/kg (7 days/week), or drug combinations in which each compound was administered at the same dose and scheduled as a single agent. Tumor sizes were measured every three days by caliper after implantation and tumor volume was calculated by the modified ellipsoidal formula: tumor volume = ½ length × width^2^. The investigators were not blinded to allocation during experiments and outcome assessment.

### NMR Spectroscopy

All NMR experiments were acquired on Bruker NEO 600 MHz spectrometer equipped with a TCI cryoprobe at 25 °C. 0.1 mM ^13^C, ^15^N- enriched PIN1 sample dissolved in pH 6.6 buffer made-up of 20 mM Potassium Phosphate, 100 mM NaSO_4_ and 10% D_2_O was used to study peptide interaction. NMR assignments of PIN1 were taken from the BMRB database (accession number 27579) and confirmed using 3D- HNCA experiment. Synthetic peptides CDH1-pS163 (comprised of CDH1 residues 161-183 with phosphorylated Ser163 and isotope labeled Pro164, LR(pS)P(^13^C, ^15^N)RKPTRKISKIPFKVLDAPE) were purchased from Pepmic. A systematic titration between PIN1 and phosphopeptide was performed by acquiring 2D- HSQC spectra. The absolute average chemical shift perturbation was calculated by using an equation, [(Δδ_H_^2^+(Δδ_N_/5)^2^)/2]^1/2^, available in software NMRFAM Sparky version 1.414.

### Experiment-guided Model

The chemical shift perturbation was interpreted as ambiguous iterative restrains used for docking a random conformation of the phosphopeptide on PIN1 (PDB: 1PIN)^51^ using HADDOCK2.2 Webserver^85^. The restrains were derived by marking two strongly perturbed PIN1 residues, R17 and W34 as active residues and three moderately perturbed residues S18, Y23 and E35 as passive residues. The peptide was assumed to be fully flexible with the phosphoserine, pS163, and its adjacent proline, P164, being the active residues that interact with PIN1. In subsequent runs, the model was refined using ambiguous distance restraints based on the interpretation of previously solved crystal structures of similar phosphopeptides bound to the WW domain of PIN1^86^.

### Proline Isomerization Study

Commercially synthesized specific ^13^C, ^15^N- P164 labeled CDH1 phosphopeptide was used to facilitate direct quantitative determination of the cis and trans proline populations. The strong ^13^C-HSQC peaks originating from Pro164 can be easily distinguished from the weak peaks due to ~1% natural abundance ^13^C present in the rest of the peptide. Two isolated sets of peaks were observed for P164. Based on the interpretation of the chemical shifts, the major peaks were assigned as trans isomer and the minor peaks were assigned as cis isomer^87^. The proline resonance assignments were further confirmed using a 2D- ^13^C-HSQCTOCSY experiment while no attempts were made to stereospecifically assign proton resonances, thus the assignment of HB2 and HB3, HG2 and HG3, and HD2 and HD3 are interchangeable. 58 *μ*M free peptide and its complex with a 4-fold molar excess of PIN1, dissolved in the above-mentioned NMR buffer were used to estimate the cis and trans isomer populations at 25°C.

### Docking model

A docking model was built to explain the interaction between PIN1 (PDB: 1PIN)^51^ and the WD40 domain of CDH1 (PDB: 4UI9_R)^52^ using the HADDOCK2.2 Webserver^85^. The docking was performed using ambiguous iterative restraints between PIN1 residues K117 to G128 and FRZ1 residues on the D-box binding interface, as observed in the anaphase-promoting complex (PDB: 4UI9)^52^. The model was refined using additional weak ambiguous distance restraints between the two canonical PIN1 residues, R119 and L122, and CDH1 residues D180, P182, E465 and L467.

### Immunofluorescence analysis

PDOX tumor tissue sections were boiled in 10 mM sodium citrate (pH 6.0), for antigen retrieval after deparaffinization. The sections were permeabilized with PBS containing 0.1%–0.5% Triton X-100 and blocked with PBS containing 5% Goat serum for 30 min RT. The primary antibodies were diluted in PBS containing 1% Goat serum (1: 200) and incubated in slides for overnight at 4°C. The slides were rinsed by PBS three times, each time for 5 min. Secondary antibodies were diluted in PBS (1:1000) and incubated for 20 min at room temperature. 20 mg/ml DAPI was used to label nuclear of cells. Slides were scanned at least three different representative areas at 20X magnification using BZ-X800 fluorescence microscope (KEYENCE).

### Quantification and statistical analysis

GraphPad Prism 9, FlowJo v10.6.2, MATLAB 2019b, PyMOL 4.60 and RStudio 4.0.2 were used to generate most charts and statistical analyses. Image J was used to quantify the relative intensity of IBs. The Database for Annotation, Visualization and Integrated Discovery (DAVID) was used to Identify enriched biological themes, particularly GO terms. Data are typically mean ± s.d. or mean ± s.e.m. Data were analyzed by unpaired two-sided t-test. Kaplan-Meier survival analysis was used for survival studies, and the groups were compared using the log-rank test. Differences of *P < 0.05, **P < 0.01, ***P < 0.001, and ****P < 0.001 were considered statistically significant. Statistical details of experiments, including statistical tests and sample sizes used, can be found in the Fig. legends. All experiments were performed on biological replicates unless otherwise specified.

## Extended Data
